# A unique cerebellar pattern of microglia activation in a mouse model of encephalopathy of prematurity

**DOI:** 10.1002/glia.24190

**Published:** 2022-05-17

**Authors:** Luisa Klein, Juliette Van Steenwinckel, Bobbi Fleiss, Till Scheuer, Christoph Bührer, Valerie Faivre, Sophie Lemoine, Corinne Blugeon, Leslie Schwendimann, Zsolt Csaba, Cindy Bokobza, Dulcie A. Vousden, Jason P. Lerch, Anthony C. Vernon, Pierre Gressens, Thomas Schmitz

**Affiliations:** ^1^ Department of Neonatology Charité University Medicine Berlin Berlin Germany; ^2^ NeuroDiderot, Inserm Université de Paris Paris France; ^3^ School of Health and Biomedical Sciences RMIT University Melbourne Victoria Australia; ^4^ Genomics Core Facility, Département de Biologie, École Normale Supérieure, Institut de Biologie de l'ENS (IBENS), CNRS, INSERM Université PSL Paris France; ^5^ Mouse Imaging Centre The Hospital for Sick Children Toronto Ontario Canada; ^6^ Department of Medical Biophysics University of Toronto Toronto Ontario Canada; ^7^ Wellcome Trust Centre for Integrative Neuroimaging University of Oxford Oxford UK; ^8^ Department of Basic and Clinical Neuroscience, Institute of Psychiatry, Psychology and Neuroscience King's College London London UK; ^9^ MRC Centre for Neurodevelopmental Disorders King's College London London UK

**Keywords:** encephalopathy of prematurity, neuroinflammation, tertiary phase

## Abstract

Preterm infants often show pathologies of the cerebellum, which are associated with impaired motor performance, lower IQ and poor language skills at school ages. Using a mouse model of inflammation‐induced encephalopathy of prematurity driven by systemic administration of pro‐inflammatory IL‐1β, we sought to uncover causes of cerebellar damage. In this model, IL‐1β is administered between postnatal day (P) 1 to day 5, a timing equivalent to the last trimester for brain development in humans. Structural MRI analysis revealed that systemic IL‐1β treatment induced specific reductions in gray and white matter volumes of the mouse cerebellar lobules I and II (5% false discovery rate [FDR]) from P15 onwards. Preceding these MRI‐detectable cerebellar volume changes, we observed damage to oligodendroglia, with reduced proliferation of OLIG2+ cells at P10 and reduced levels of the myelin proteins myelin basic protein (MBP) and myelin‐associated glycoprotein (MAG) at P10 and P15. Increased density of IBA1+ cerebellar microglia were observed both at P5 and P45, with evidence for increased microglial proliferation at P5 and P10. Comparison of the transcriptome of microglia isolated from P5 cerebellums and cerebrums revealed significant enrichment of pro‐inflammatory markers in microglia from both regions, but cerebellar microglia displayed a unique type I interferon signaling dysregulation. Collectively, these data suggest that perinatal inflammation driven by systemic IL‐1β leads to specific cerebellar volume deficits, which likely reflect oligodendrocyte pathology downstream of microglial activation. Further studies are now required to confirm the potential of protective strategies aimed at preventing sustained type I interferon signaling driven by cerebellar microglia as an important therapeutic target.

## Main Points

- There is sustained activation of microglia in the developing cerebellum after systemic inflammation, being accompanied by delayed oligodendrocyte maturation and myelination.
- RNAseq on sorted microglia reveals interferon‐mediated pathway upregulation.

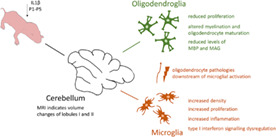

## 1 INTRODUCTION

Babies born preterm (at less than 37 weeks of gestation) have a pronounced vulnerability to cerebellar injury (Chang et al., 2000; Volpe, 2009a), which remains under‐acknowledged, and as such, cerebellar pathologies are likely to be underdiagnosed (Hintz et al., 2015). Approximately 15 million babies are born premature every year (~10% of all births) (Howson et al., 2013). Furthermore, damage of the immature cerebellum is increasingly linked to cognitive impairment including specific deficiencies in executive function, deficits of attention, and increased risk for psychiatric disorders with a neurodevelopmental origin such as autism spectrum disorder (ASD) (Baillieux et al., 2008; Bouyssi‐Kobar et al., 2016; Fatemi et al., 2012; Johnson & Marlow, 2011; Koziol et al., 2014; Limperopoulos et al., 2007; Parker et al., 2008) and schizophrenia (Botellero et al., 2016). Hence, there is a significant need to understand how cerebellar maldevelopment and injury develop in these infants.

In this context, clinical studies and animal models provide evidence that exposure to systemic inflammation in the fetal and neonatal periods plays a key role in driving brain injury and cerebellar maldevelopment (Bartha et al., 2004; Nelson et al., 2003; Schmitz et al., 2014; Wu et al., 2000). Specifically, brain injury occurring in preterm born infants is termed encephalopathy of prematurity (EoP), and it is characterized by neuroinflammation, oligodendrocyte maturation arrest and hypomyelination, axonopathy, reduced white matter fractional anisotropy and cortical volume determined by magnetic resonance imaging (MRI), all of which precede overt clinical symptoms such as cognitive deficits (Volpe, 2009b). These damaging effects of systemic inflammation may be linked to two main processes. First, that systemic inflammation causes preterm birth, thus depriving the infant of trophic support including from the placenta and exposing it to the noxious extra‐uterine environment (Volpe, 2009c). Second, that systemic inflammation prevents glial cells from performing important brain building functions and instead forces them into a damaging inflammatory reactivity (Malaeb & Dammann, 2009; Yanni et al., 2017). A common source of damaging systemic inflammation is chorioamnionitis, which has specifically been identified as a risk factor for a diagnosis of cerebral palsy (Malaeb & Dammann, 2009; Yanni et al., 2017) and ASD (Bolduc et al., 2012; Brossard‐Racine et al., 2015; Limperopoulos et al., 2008). Although strong inflammatory insults such as chorioamnionitis are known drivers of brain changes, importantly the presence of subtle, low‐grade perinatal/postnatal inflammation may also occur, for example in response to mechanical ventilation (Malaeb & Dammann, 2009; Volpe, 2009c), medical interventions and medication (Favrais et al., 2011), all of which have been shown to cause brain injury in experimental models (Sobotka et al., 2016). To better understand the mechanistic links between mild systemic inflammation and EoP, it is critical to increase our knowledge of the responses of the brain's resident innate immune cells, microglia, to the aforementioned inflammatory triggers, since evidence suggests these cells are a key mediator of brain injury in models of EoP (Baud et al., 2004; Dommergues et al., 2003; Krishnan et al., 2017; Van Steenwinckel et al., 2019) and in human causes of EoP (Verney et al., 2010, 2012). Further supporting this view, typically functioning microglia play critical roles in the normal development of the brain, underlined by their production of growth factors, such as insulin‐like growth factor 1 (IGF1) (Suh et al., 2013), brain‐derived neurotrophic factor (BDNF) (Nakajima et al., 2001) and transforming growth factor beta (TGFβ) (Welser‐Alves & Milner, 2013), that promote and regulate proliferation and maturation of immature neurons and oligodendrocytes (Peferoen et al., 2014; von Bernhardi et al., 2016). Finally, microglia are also involved in regulating neuronal death and shaping connectivity by regulating synaptic pruning during brain development (Bessis et al., 2007), including in the cerebellum (Nakayama et al., 2018).

It may therefore be hypothesized that one mechanism by which early exposure to perinatal systemic inflammation would have adverse effects on the developing cerebellum is through microglia. To test this hypothesis, we used an established mouse model of EoP triggered by systemic exposure to the pro‐inflammatory cytokine interleukin‐1β (IL‐1β) during a developmental stage of the mouse brain (postnatal day 1–5) that corresponds to a window of vulnerability for human preterm birth (23–32 weeks' gestation). We have already demonstrated using this mouse model that perinatal inflammation driven by systemic IL‐1β exposure disrupts the macroscale development of mouse brain white matter pathways, as measured by diffusion tensor imaging (DTI) (Favrais et al., 2011). At the cellular level, we have provided evidence in the mouse forebrain that this is a consequence of oligodendroglial dysmaturation (Favrais et al., 2011; Krishnan et al., 2017; Schang et al., 2014), which is specifically triggered by microglial reactivity (Favrais et al., 2011) and can be prevented or diminished by inhibition of microglia (Van Steenwinckel et al., 2019). The impact of systemic IL‐1β on cerebellar development in this mouse model and the underlying cellular causes have however yet to be investigated. Using structural MRI, we observed a strikingly specific effect on cerebellar development, characterized by reduced cerebellar gray and white matter volumes. Guided by these data, we carried out focused in vivo and ex vivo studies on the cerebellar oligodendrocytes and microglia to link these macroscale changes to their cellular and molecular correlates. The results indicate that in addition to oligodendrocyte damage and myelin deficits caused by systemic inflammation, there is a persistent increase in microglial density and amoeboid “activated” morphology in the cerebellum accompanied by a cerebellum‐specific transcriptomic pattern of microglial reactivity driven by interferon type 1. The results of this study highlight brain region specific consequences of perinatal systemic inflammation on the developmental program of oligodendrocytes and microglia in the cerebellum and uncover a novel pathway regulating this region‐specific outcome.

## 2 METHODS

Experimental protocols were approved by the institutional guidelines of the Institut National de la Santé et de la Recherche Medicale (Inserm, France) (Approval 2012‐15/676‐0079 and 2012‐15/676‐0083), the Ethics Committee and the services of the French Ministry in charge of Higher Education and Research according to the directive 2010/63/EU of the European Parliament (#9286‐2016090617132750).

### 2.1 Mouse model of encephalopathy of prematurity

Experiments were performed using OF1 strain mice (Charles River, France). Systemic perinatal inflammation was induced as previously described (Favrais et al., 2011; Krishnan et al., 2017; Schang et al., 2014; Van Steenwinckel et al., 2019). Briefly, a 5 μl volume of phosphate‐buffered saline (PBS) containing 10 μg/kg/injection of recombinant mouse IL‐1β (Miltenyi Biotec, Bergisch Gladbach, Germany) or of PBS alone (control) was injected intraperitoneally (i.p.) twice a day on days P1 to P4 and once a day on day P5. The timing of IL‐1β injections (P1–P5) was chosen to mimic a chronic exposure to circulating cytokines at a developmental stage of the brain corresponding to human preterm birth (23–32‐weeks gestational age). In previous studies using this model, we provide evidence that IL‐1β injections between P1 and P5, compared to PBS injection had no effect on mortality, or body weight at ages P1, P5, and P30 (Favrais et al., 2011). By contrast, there is a moderate decrease in minute ventilation and also an increase in apnea duration in IL‐1β injected animals (while total duration of apneas were low). These respiratory changes were accompanied by a minor increase in blood HCO3‐ but had no significant impact on blood pH, PCO2, PO2, hemoglobin, or heart rate (Favrais et al., 2011), supporting the idea of a mild inflammatory reaction without systemic inflammatory symptoms (Favrais et al., 2011). Prior studies in this mouse model also suggest that male pups show a more profound phenotype of white matter damage in response to systemic IL‐1β, as compared to females(Krishnan et al., 2017), mimicking the higher vulnerability of preterm born males to poor neurodevelopmental outcomes (Ball et al., 2017; Johnston & Hagberg, 2007). Based on these data, we only used male pups in the present study. To facilitate analysis of cellular proliferation, male pups received systemic injections of bromo‐deoxyuridine (BrdU; BD Biosciences, San Jose, CA) (10 mg/kg per day, P1–P4) in conjunction with either IL‐1β or PBS‐injections.

### 2.2 MRI

#### 2.2.1 Brain perfusion for ex vivo MRI

There are important trade‐offs to consider when choosing between in vivo and ex vivo MR imaging. The former enables longitudinal imaging, with potentially greater statistical power, whilst the latter permits acquisition of higher resolution images with increased sensitivity, particularly when contrast enhancement is used (Holmes et al., 2017; Lerch et al., 2012). For this study we opted for ex vivo MR imaging with contrast enhancement, first to maximize our ability to detect subtle differences in neuroanatomy and second for logistical ease since the animal and MR imaging facilities were not co‐located. Two cohorts of male pups sampled randomly from five independent litters were sacrificed at P15 (PBS, *n* = 9; IL‐1β, *n* = 9) and P60 (PBS, *n* = 8; IL‐1β, *n* = 11) for ex vivo structural MRI. Littermates from these mice were assessed at P10 with qRT‐PCR to ascertain they had the requisite oligodendrocyte maturation delay that is characteristic of the model as described (Favrais et al., 2011). Details of the perfusion protocol have been described at length elsewhere (Cahill et al., 2012). Briefly, mice were anesthetized with pentobarbital and then transcardially perfused with 30 ml of 0.1 M PBS containing 10 U/ml heparin and 2 mM Prohance (a gadolinium based contrast agent; Bracco Inc., Bucks, UK), followed by 30 ml of 4% paraformaldehyde (PFA) also containing 2 mM Prohance. Post‐perfusion mice were decapitated and the skin, ears and lower jaw removed, but the brain was left inside the skull to minimize distortions or deformations from dissection. Fixed brain tissues were then incubated in 4% PFA + Prohance overnight followed by incubation in 0.1 M PBS containing 2 mM Prohance and 0.05% (w/v) sodium azide to allow tissue rehydration. Acquisition of ex vivo structural MR images from the fixed and rehydrated brains therefore took place 14 days after the initial perfusion.

#### 2.2.2 Structural MRI acquisition

A 7 T horizontal small‐bore magnet (Agilent Technologies Inc, Santa Clara, USA) and a quadrature volume radiofrequency coil (39 mm internal diameter, Rapid Biomedical GmbH) were used for all ex vivo structural MRI acquisitions. Rehydrated fixed brain samples were placed securely one a time in an MR‐compatible holder immersed in proton‐free susceptibility matching fluid (Fluorinert™ FC‐70; Sigma‐Aldrich, St‐Louis, MO). In order to assess volume differences throughout the mouse brain, a T2‐weighted 3D Fast Spin‐Echo (FSE) sequence was used, with the following sequence parameters: TR = 1000 ms, echo train length (ETL) = 16, echo spacing = 5.68 ms, TE_eff_ = 34 ms, field of view (FOV) = 19.2 mm × 19.2 mm × 19.2 mm and a matrix size of 192 × 192 × 192, yielding an isotropic (3D) resolution of 100 μm. Total imaging time for each brain was 38 min.

#### 2.2.3 MR image registration and statistical analysis

Image registration and analysis were carried out at the Mouse Imaging Centre (MICe) at the University of Toronto, ON, Canada. Deformation based morphometry (DBM) was used to analyze volume differences between PBS and IL‐1β‐exposed mice at P15 and P60. DBM is performed by registering the individual T2‐weighted MR images from each mouse brain together via a series of linear (6, then 12‐parameter fit) and non‐linear registration steps using a fully automated pipeline (Lau et al., 2008; Vousden et al., 2018). Post‐registration, all scans are resampled with an appropriate transform and averaged to create a population atlas, which represents the average anatomy of the mice in the study. Registration was performed using a combination of mni_autoreg tools (Collins et al., 1994) and advanced normalization tools (Avants et al., 2008, 2011). The registration brings all the scans into alignment via deformation in an unbiased manner, allowing the experimenter to then analyze the deformations required to take each individual mouse's anatomy into a final atlas space. The Jacobian determinants of the deformation fields are utilized as the measures of apparent volume change on a per voxel basis, corrected for inter‐individual mouse differences in total brain volume (Vernon et al., 2014). Regional volume differences are then calculated by warping a pre‐existing classified MRI atlas onto the population atlas, permitting the calculation of regional volumes across the brain (Dorr et al., 2008). This MRI atlas includes 159 different structures incorporating three separate pre‐existing atlases: (1) 62 different structures throughout the mouse brain including subcortical white and gray matter structures, corpus callosum, striatum, and thalamus (Dorr et al., 2008) (2) further divides the cerebellum into its various regions, individual lobules, white and gray matter, and the deep cerebellar nuclei (Steadman et al., 2014); and (3) divides the cortex into 64 different regions, including areas of the cingulate cortex and primary motor and somatosensory cortices (Ullmann et al., 2013). The total brain volume is also included. Differences in brain volume were analyzed using atlas‐derived regional brain volumes via mixed linear modeling with age as within group factor (P15 and P60), treatment (PBS or IL‐1β) as between group factor and age × treatment interactions (Lau et al., 2008). Multiple comparisons were controlled for using the false discovery rate (FDR) at 5% (*q* < 0.05) (Genovese et al., 2002).

### 2.3 Tissue preparation for cellular and molecular analyses

Brain tissue samples were collected at P3, P5, P10, P45, P60, and P150 (Figure S1). For molecular studies, mouse cerebellums were snap‐frozen in liquid nitrogen and stored at −80°C until further analysis. For immunohistochemical studies, mice were perfused with 0.1 M PBS followed by 4% paraformaldehyde. The brains were then postfixed at 4°C for 3 days, embedded in paraffin, and processed for histological staining. Sagittal paraffin sections of 10 μm were obtained and mounted onto Super Frost plus™‐coated slides (R. Langenbrinck, Emmendingen, Germany). Sections were deparaffinized using Roti‐Histol Solution (Carl Roth, Karlsruhe, Germany) twice for 10 min each and rehydrated in descendant ethanol concentrations (from 100%, 100%, 90%, 80%, 70%; 3 min each) and finally distilled water (3 min). For immunostainings, sagittal sections were selected according to sagittal positions 186–195 of the Allen mouse reference atlas (developing brain) (https://mouse.brain-map.org/static/atlas).

### 2.4 Protein extraction

Snap‐frozen whole mouse cerebellums were homogenized in radioimmunoprecipitation assay buffer (RIPA Buffer; Sigma–Aldrich) with complete Mini EDTA‐free Protease Inhibitor Cocktail Tablets (Roche Diagnostics, Mannheim, Germany). The homogenate was centrifuged at 13,000*g* (4°C) for 20 min before collecting the supernatant. Protein concentrations were determined using the Bicinchoninic Acid Protein Assay kit (Pierce/Thermo Scientific, Rockford, IL).

### 2.5 Immunoblotting

Immunoblotting was performed as previously described (Endesfelder et al., 2014). Briefly, samples were diluted in Laemmli sample loading buffer (Bio‐Rad, Munich, Germany) denatured (95°C for 5 min) and protein extract (20 μg per lane) were cooled on ice, separated electrophoretically on 10%–12% Mini‐PROTEAN TGX precast gels (Bio‐Rad), and transferred onto nitrocellulose membrane (0.2 μm pore; Bio‐Rad) using a semidry electrotransfer unit at 15 V for 5 min. By staining the membranes with Ponceau S solution (Fluka, Buchs, Switzerland) equal loading and transfer of proteins was confirmed. After blocking the membranes with 5% bovine serum albumin (BSA) in Tris‐buffered PBS/0.1% Tween 20 for 1 h at room temperature they were incubated overnight at 4°C with the following antibodies: mouse monoclonal anti‐myelin‐associated glycoprotein (MAG, 63 and 69 kDa; 1:500; Abcam, Cambridge, UK), rabbit polyclonal anti‐myelin oligodendrocyte protein (MOG, 27 kDa; 1:1000; Abcam), mouse monoclonal anti‐myelin basic protein (MBP, 18 and 24 kDa; 1:1000; Covance, Princeton, NJ) or mouse monoclonal anti‐β‐actin (42 kDa; 1:5000; Sigma Aldrich), respectively. Secondary incubations were performed with horseradish peroxidase‐linked polyclonal goat anti‐rabbit (1:5000; Dako, Glostrup, Denmark) or polyclonal rabbit anti‐mouse (1:5000; Dako) antibodies. Positive signals were visualized using enhanced chemiluminescence (Amersham Biosciences, Freiburg, Germany) and quantified using a ChemiDoc XRS+ system and the software Image Lab (Bio‐Rad). To ensure the equal loading and accuracy of changes in protein abundance, the protein levels were normalized to β‐actin.

### 2.6 Immunostaining

To increase cell permeability mouse brain tissue sections on slides were microwaved for 10 min at 600 W in citrate buffer (pH 6.0) and afterwards left for 30 min at room temperature. For staining with BrdU it was necessary to apply 0.1 mM hydrochloric acid for 20 min and then neutralize it with sodium borate buffer. Slides were cooled to room temperature and washed using PBS solution before blocking non‐specific binding for 1 h at room temperature with blocking solution (1% BSA, 10% normal goat serum (NGS), 0.05% Tween‐20 in PBS). Primary antibodies (Table S1a) were diluted in DAKO Antibody diluent (Dako Deutschland, Hamburg, Germany) and subsequently incubated at 4°C for 24–48 h. Secondary fluorescent antibodies (Table S1b) were diluted 1:200 in Dako Antibody diluent and incubated at room temperature for 1 h. Sections were counterstained with 4,6‐diamidino‐2‐phenylindole (DAPI, 10 ng/ml, Sigma Aldrich, diluted 1:2000 in PBS, incubation time 10 min). Slides were mounted using mounting medium (Vectashield HardSet Mounting Media, Vector Laboratories, Burlingame, CA).

After immunofluorescent stainings, the 10 μm cerebellar tissue slices were viewed using a Keyence BZ‐9000 microscope at ×20 magnifications. Tissue section was selected by approximating 0.2–0.4 mm lateral position of Paxinos and Watson mouse brain atlas (2nd Edition, Academic Press, 2001). Images were taken from the proximal/central parts of in lobules I‐VI in, which in rodents start being myelinated at postnatal ages, similar to methods previously described in newborn rats (Scheuer et al., 2015). The average of three images was used as resulting number for one N. Confocal Z‐stacks were created from each section and fused using BZII analyzer software (Keyence, Osaka, Japan). After minimal adaptation of contrasts to facilitate cell counting using Adobe® Photoshop CS6 Extended version 13.0, the stained cells were counted with the operator blinded to the treatment of the mice. The cell counts were used to calculate average values and statistical analysis with the help of GraphPad PRISM™ software (see Section 2.10).

### 2.7 Cell sorting and RNA extraction from isolated cells

Cerebrum and cerebellum at P5 were collected for cell dissociation using Neural tissue Dissociation (Miltenyi Biotec) kit with papain and double cell isolation using magnetic coupled antibodies anti‐CD11B (Microglia) according to the manufacturer's protocol (Miltenyi Biotec) and as previously described (Krishnan et al., 2017; Van Steenwinckel et al., 2019). Cells were pelleted and conserved at −80°C. RNA from CD11B+ cells from brains of mice exposed to IL‐1β or PBS at P5 were extracted (RNA XS plus, Macherey‐Nagel, Dueren, Germany).

### 2.8 RNA sequencing and bioinformatics analysis

Library preparation and Illumina sequencing were performed at the Ecole Normale Supérieure genomic core facility (Paris, France). Messenger (polyA+) RNAs were purified from 100 ng of total RNA using oligo(dT). Libraries were prepared using the strand specific RNA‐Seq library preparation TruSeq Stranded mRNA kit (Illumina, Évry‐Courcouronnes, France). Libraries were multiplexed by 8 on 4 high‐output flow cells. Four 75 bp single read sequencing were performed on a NextSeq 500 (Illumina). The analyses were performed using the Eoulsan pipeline (Jourdren et al., 2012), including read filtering, mapping, alignment filtering, read quantification, normalization, and differential analysis: Before mapping, poly N read tails were trimmed, reads ≤40 bases were removed, and reads with quality mean ≤30 were discarded. Reads were then aligned against the Mus musculus genome from Ensembl version 91 using STAR (version 2.6.1b) (Dobin et al., 2013). Alignments from reads matching more than once on the reference genome were removed using Java version of samtools (Li et al., 2009). To compute gene expression, Mus musculus GTF genome annotation version 91 from Ensembl database was used. All overlapping regions between alignments and referenced exons were counted using HTSeq‐count 0.5.3 (Anders et al., 2015) and then aggregated to each referenced gene. The sample counts were normalized using DESeq2 1.8.1 (Love et al., 2014). Statistical treatments and differential analyses were also performed using DESeq2 1.8.1. Heatmap and hierarchical clustering were realized with one minus Pearson correlation and was generated using Morpheus (https://software.broadinstitute.org/morpheus/). Significant differentially expressed (DE) genes under IL‐1β condition in CD11B+ cells were identified with Benjamini and Hochberg adjusted *p*‐values <.05. Functional analyses were done using DAVID 6.8 (https://david.ncifcrf.gov). Analysis of predicted protein interactions was undertaken using STRING (https://string-db.org) with an interaction stringency of 0.400 (medium confidence) and first and second shell interactions set to none to allow the significance of the interactions to be accurately assessed. To access WikiPathways (https://www.wikipathways.org) we used the Enrichr tool (https://maayanlab.cloud/Enrichr/;Kuleshov). Significant representative GO terms, STRING local network clusters and Wikigene pathways (mouse) were identified with Benjamini and Hochberg adjusted *p*‐values <.05.

### 2.9 Fluorescence activated cell sorting (FACS) analysis

Cerebrum and cerebellum at P5 were collected for cell dissociation using Neural Tissue Dissociation (Miltenyi Biotec). The cells were counted (Nucleocounter NC‐200, Chemometec, Allerod, Denmark) and re‐suspended at 10 × 10^6^ cells/ml in FACs buffer (Dulbecco's Phosphate Buffer Saline (Gibco Life Technologies, Paisly, UK), 2 mM EDTA (Sigma Aldrich, Saint Louis, MO), 0.5% BSA (Miltenyi Biotec). After Fc blocking (BD Biosciences, Le Pont De Claix, France), cells were incubated with viability probe (FVS780, BD Biosciences) and fluorophore‐conjugated antibodies against mouse CD45, CD11B (CD11B BV421, clone MI/70, CD45BV510, clone 30‐F11; Sony Biotechnology, San Jose, CA), CD18 (CD18 APC, clone C71/16; BD Biosciences, Le Pont De Claix, France) or their corresponding control isotypes (Sony Biotechnology and BD Biosciences), at concentrations recommended by the manufacturers or calculated after titration. Cells were washed with FACs buffer, resuspended in PBS and FACs analysis was done within 24 h. After doublets and dead cells exclusion based respectively on morphological parameters and FVS780 staining, gating strategy selected microglia as live/CD11B^+^/CD45^int^. Expression of CD18 was analyzed in microglia. Myeloid cells (including polymorphonuclear neutrophils, monocytes, and macrophages) were defined as CD11B^+^/CD45^high^. For oligodendrocyte marker O4 (O4)/platelet derived growth factor receptor alpha (PDGFRa) analysis, only P5 cerebellum cells were analyzed. After Fc blocking (BD Biosciences), cells were incubated with viability probe (FVS780, BD Biosciences) and fluorophore‐conjugated antibodies against mouse O4 (O4 VioBright 515, Miltenyi Biotech) and PDGFRa (CD140a‐PE, Miltenyi Biotech). One cerebellum ex vivo was used as *n* = 1 for individual FACS with cell count.

### 2.10 Statistical analysis of neuropathology analyses

Statistical data were analyzed using GraphPad PRISM™ (GraphPad Inc., La Jolla, CA). First, a Grubb's outlier test was performed for each subgroup and outliers identified were removed from further analyses. Subsequently, Kolmogorov–Smirnov tests were used to check for normal distribution and, accordingly, either a *t*‐test (for normally distributed samples) or a nonparametric Mann–Whitney‐*U* test (for non‐normally distributed samples) were performed. A *p*‐value of <.05 was considered significant (*<.05; **<.01; ***<.001). The quantitative data are presented in the figures as a scatter dot‐plot with median for each intervention group. Values are given rounded to two decimal places as median ± standard deviation (SD). For western blot, immunohistochemistry quantification and FACS analysis of O4, PDGFRa, CD11B+CD45lo and CD11B+CD45hi cells, normal distribution of each data set was verify using Shapiro–Wilk test. Two‐tailed Student *t*‐test were realized to compare PBS and IL‐1β condition in each data set. For quantification of fluorescence intensity of CD11B and CD118, normal distribution of each data set was verify using Shapiro–Wilk test and Two‐way ANOVA with Tukey post‐hoc test were used to compare PBS and IL‐1β treatments but also cerebrum and cerebellum in each data. Significant modulation of microglial markers expression level from RNA‐sequencing data were identified with Benjamini and Hochberg adjusted *p*‐values <.05. (+*p* < .05; ++*p* < .01; +++*p* < .001).

## 3 RESULTS

### 3.1 Systemic perinatal inflammation specifically reduces total and regional mouse brain volumes with a specific effect on the cerebellum

**FIGURE 1:**
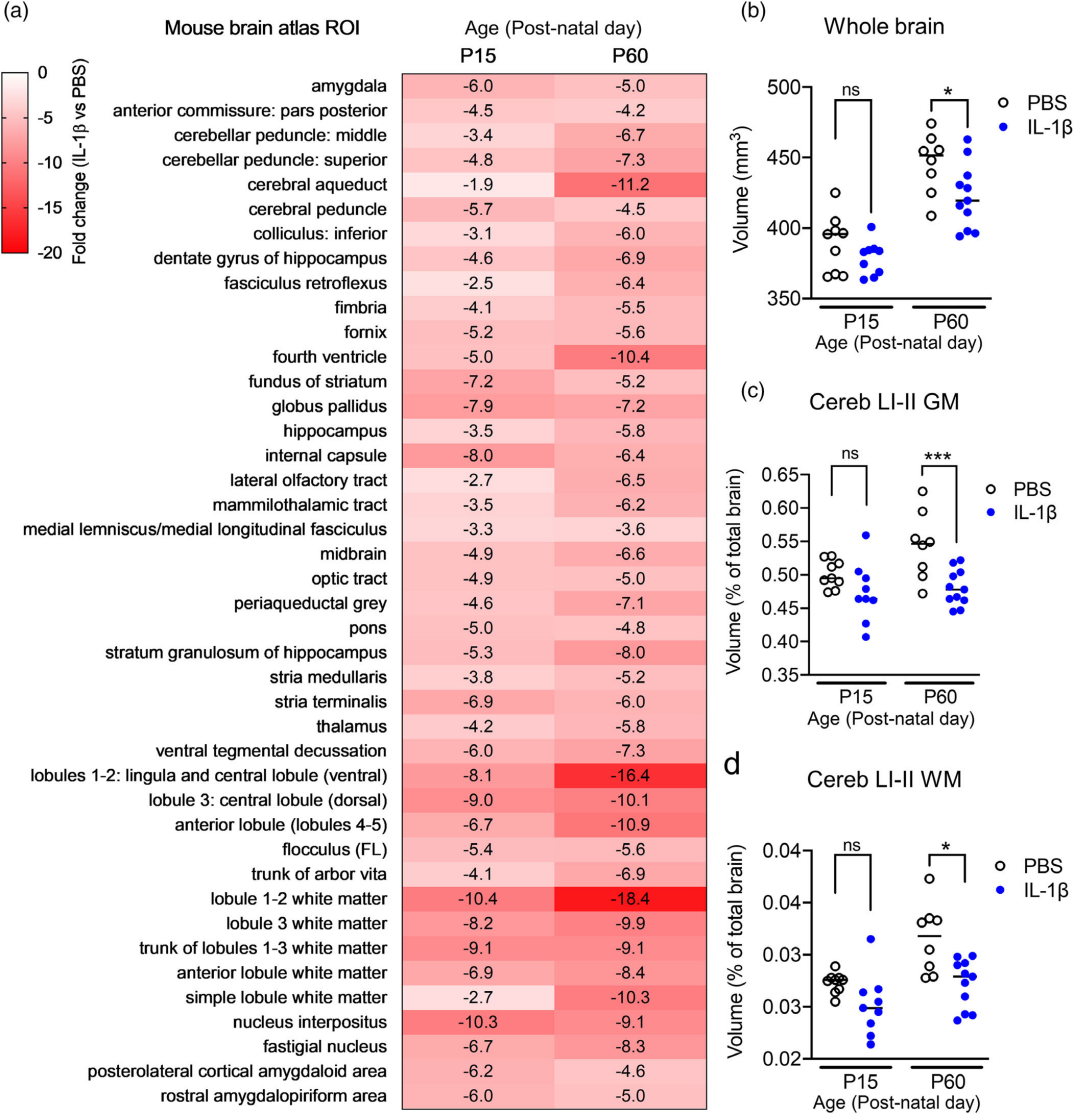
Impact of systemic inflammation on brain volumes. (a) Heat map to illustrate the % change in absolute volumes (mm^3^) between IL‐1β‐exposed mice relative to PBS controls at P15 and P60, derived from atlas based segmentation. Only brain regions that were significantly different after multiple comparisons correction (5% FDR) are shown. (b) Whole brain volume is however decreased in IL‐1β‐exposed mice relative to PBS controls at P60, but not P15. Considering relative volumes therefore to account for possible scaling effects (regional volumes in mm^3^ expressed as a % of total brain volume) only two brain regions remained statistically significant between the groups after FDR‐correction, these being. (c) The relative volumes of the cerebellar gray matter (GM) and (d) cerebellar white matter (WM) in lobules I‐II. Data points represent individual mice in each group with the horizontal line indicating the group mean. *p < .05; ***p < .001 PBS versus IL‐1β‐exposed mice, post‐hoc analysis corrected for multiple comparisons (2‐step FDR method at 5%). FDR, false discovery rate; ns, not significant; PBS, phosphate buffered saline

Total mouse brain volume increased as a function of age between P15 and P60 (*F*(1, 33) = 59.4; *p* < .0001) (Figure 1a). Whilst systemic IL‐1β exposure also impacted on total mouse brain volume (*F*(1, 33) = 6.7; *p* < .01), this did not interact with age (*F*(1, 33) = 0.99; *p* = .34). Post‐hoc testing on the main effect of treatment revealed a reduction in mouse total brain volume at P60 (−5.3%; *p* = .02; *q* = 0.02), but not at P15 (−2.7%; *p* = .27; *q* = 0.14) in mice exposed to systemic IL‐1β relative to controls (Figure 1a).

We next tested for any effects of systemic perinatal inflammation on regional mouse brain volumes, using atlas‐based segmentation (ABS) (Figure 1b, Figure S2). Using absolute volumes (mm^3^) as the dependent variable, after correction for multiple comparisons 64% (102/159) of mouse brain atlas regions of interest (ROI) were statistically significantly affected by increasing postnatal age across both treatment groups. In total, 27% (43/159) of atlas ROIs were also statistically significantly different as a function of IL‐1β exposure, relative to PBS‐exposed mice (Table S2). Each of these 43 mouse brain regions were smaller in IL‐1β‐exposed mice, relative to PBS‐exposed mice at both P15 and P60 (Figure 1b). Whilst these areas covered a range of cortical and sub‐cortical regions as well as ventricular structures, the effects of systemic perinatal inflammation were particularly concentrated in the cerebellar gray and white matter, which displayed the largest apparent volume reductions (% change; Table S2). Across all mouse brain atlas ROIs however, there were no statistically significant age x treatment interactions (Table S2).

Absolute volume analysis however, cannot determine whether brain regions were smaller but normally scaled, or if any individual brain regions were abnormally scaled relative to whole brain volume change following systemic inflammation (Clipperton‐Allen et al., 2019). Given that mouse whole brain volume differed between treatment groups at P60, we next tested for any effect of such scaling on the volumes of individual brain regions between groups, by calculating the relative volume of each brain region ([(brain region volume)/(whole brain volume) × 100] (Doostdar et al., 2019). Using relative volumes as the dependent variable revealed that 74% (118/159) of mouse atlas brain regions were altered with increasing post‐natal age across both treatment groups (Table S2). In contrast to the absolute volume data however, only 1% (2/159) of atlas ROIs were statistically significantly different in IL‐1β‐exposed mice relative to PBS‐exposed mice (FDR *q* < 0.05), these being the gray and white matter ROIs for cerebellar lobules I‐II (Figure 1c,d; Table S2). Of note, these regions also showed the largest % changes in the absolute volume dataset (Figure 1b; Table S2). Post‐hoc testing revealed that these changes in the lobule I‐II gray matter were statistically significant at P60, but not P15 (Figure 1c). By contrast, the difference was significant at both time‐points for the cerebellar white matter, suggestive of differential developmental timing on the effect of IL‐1β on cerebellum gray and white matter volumes (Figure 1c,d). There were however no statistically significant age x treatment interactions after correction for multiple comparisons (5% FDR). Collectively, these data suggest that systemic perinatal inflammation induces a global effect on mouse brain volume, but controlling for this suggests a more selective alteration of cerebellar white matter growth from at least P15 onwards, which is maintained at least until P60, with gray matter effects becoming apparent from P60 onwards.

### 3.2 Systemic inflammation altered myelination and oligodendrocyte maturation in the cerebellum

**FIGURE 2:**
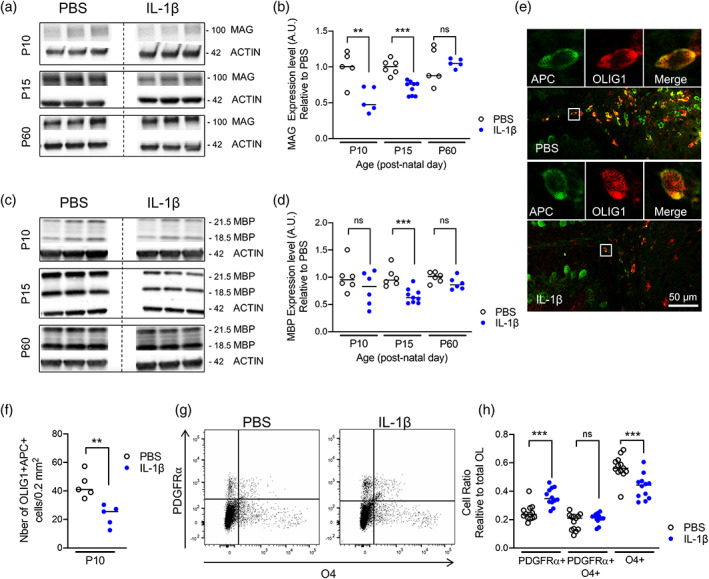
Impact of systemic inflammation on myelin and oligodendroglial maturation. (a–d) Western blotting of MAG (a and b) and MBP (c and d) of P10, P15, and P60 cerebellar tissues from PBS versus IL‐1β‐exposed mice (**p < .01, ***p < .001; *t* test). (e and f) Representative micrographs of APC+ (green) and OLIG1+ (red) cells at P10 in cerebellar slices from PBS versus IL‐1β‐exposed pups (e; scale bar = 50 μm) and corresponding quantification (f; **p < .01, *t* test). Tissue section was selected for microscopy imaging by approximating 0.2–0.4 mm lateral position of Paxinos and Watson mouse brain atlas (2nd Edition, Academic Press, 2001). (g and h) Representative scatter plots of the FACS analysis of total numbers of PDGFRa+/O4+ oligodendrocytes in P10 cerebellum from PBS or IL‐1β‐exposed pups and corresponding quantification (f; ***p < .001). Shapiro–Wilk test was used to verify normal distribution of each data set. Two‐tailed Student *t*‐tests were realized on data. MAG, myelin‐associated glycoprotein; MBP; myelin basic protein; PBS, phosphate buffered saline

Guided by the aforementioned structural MRI data, we next carried out focused post‐mortem investigations in the cerebellum to establish the cellular correlates of the macroscale volume changes in this region. In line with our hypothesis and the evidence suggesting that white matter volume changes proceed those in gray matter, we focused initially on myelin and oligodendrocytes. Protein levels of myelin basic protein (MBP) and myelin‐associated glycoprotein (MAG) at P10, P15, and P60 were measured via western blotting using whole mouse cerebellum lysates (Figure 2, Figure S3). At P10 and P15, (but not P60) there were significant reductions in the levels of MAG (median ± standard deviation; P10 PBS: 1.00 ± 0.22 vs. IL‐1β: 0.47 ± 0.17, *p* < .01, *n* = 5; P15 PBS: 1.00 ± 0.10 vs. IL‐1β: 0.76 ± 0.09, *p* < .001, *n* = 6–9; P60 PBS: 0.88 ± 0.26 vs. IL‐1β: 1.05 ± 0.06, *p* = .66, *n* = 5; Figure 2a/b) in the mice exposed to systemic inflammation. At P15 there were marked reductions in MBP expression, in comparison to control litters although MBP levels were visibly numerically lower at each age tested (median ± standard deviation; P10 PBS: 0.95 ± 0.27 vs. IL‐1β: 0.83 ± 0.32, *p* = .24, *n* = 6; P15 PBS: 0.95 ± 0.17 vs. IL‐1β: 0.62 ± 0.13, *p* < .001, *n* = 6–9; P60 PBS: 1.01 ± 0.09 vs. IL‐1β: 0.86 ± 0.11, *p* = .09, *n* = 6; Figure 2c/d). Consistent with these data, immunohistochemical analyses revealed that the numbers of oligodendrocytes co‐labeled with the oligodendrocyte marker adenomatous polyposis coli (APC) and the oligodendrocyte transcription factor 1 (OLIG1) were significantly lower at P10 in mice exposed to systemic inflammation as compared to controls (median ± standard deviation; PBS: 82.5 ± 4.78 vs. IL‐1β: 56.67 ± 8.62, *p* < .01, *n* = 5; Figure 2e/f). Of note, there were no significant differences in the density of OLIG2+ cells/ 0.2 mm^2^ between the two experimental groups (median ± standard deviation; P5 PBS: 101.5 ± 30.34 vs. IL‐1β: 83.5 ± 32.06, *p* > .05, *n* = 5; P10 PBS: 117.7 ± 34.73 vs. IL‐1β: 116.2 ± 24.38, *p* > .05, *n* = 5; Figure S3a). We then sought to determine if the reduction in mature oligodendrocytes could be explained by increased levels of cell death and/or modification of progenitor pools. We assessed cerebellum cell death at P3, P5, and P10 and found only three to six CASP3+ cells (cleaved caspase 3, apoptotic cell death marker) per 0.2 mm^2^ at P3 and P5, and three or less at P10, with no detectable impact of IL‐1β exposure (absolute cell count CASP3+: P3 *p* = .44; P5 *p* = .03, P10 *p* = 1; OLIG2+ CASP3+ cells: P3 *p* = .37; P5 *p* = .03; P10 *p* = 1; Figure S4).

We then explored the effect of systemic inflammation on oligodendroglial lineage progression using FACS at P5 to determine the numbers of PDGFR+/O4‐ OPCs, PDGFR+/O4+ intermediate precursor/immature oligodendrocytes, and PDGFR‐/O4+ immature oligodendrocytes after perinatal exposure to IL‐1β compared to controls injected with PBS. A clear increase in PDGFR+/O4‐ OPC cell number was noted in the cerebellum of pups with systemic inflammation in comparison to control pups (median ± standard deviation; PDGFR+/O4‐ PBS: 0.24 ± 0.06 vs. IL‐1β: 0.35 ± 0.06, *p* < .001). At the same time, numbers of immature oligodendrocytes were reduced in pups after inflammatory exposure when compared to controls (median ± SD; PDGFR‐/O4+ PBS: 0.56 ± 0.08 vs. IL‐1β: 0.44 ± 0.09, *p* < .001). The population of PDGFR+O4+ intermediate stage cells was not altered (median ± SD; PDGFR+/O4+ PBS: 0.21 ± 0.06 vs. IL‐1β: 0.22 ± 0.04, *p* > .05) (*n* = 12–13; Figure 2g/h). Collectively, these data indicate that MRI volume changes are driven, at least in part, by reduced myelin synthesis at P10 and P15 and by the loss of immature and mature oligodendroglia with a shift toward higher numbers of OPCs in the cerebellum.

### 3.3 Cerebellar oligodendrocyte proliferation was inhibited by systemic inflammation

**FIGURE 3:**
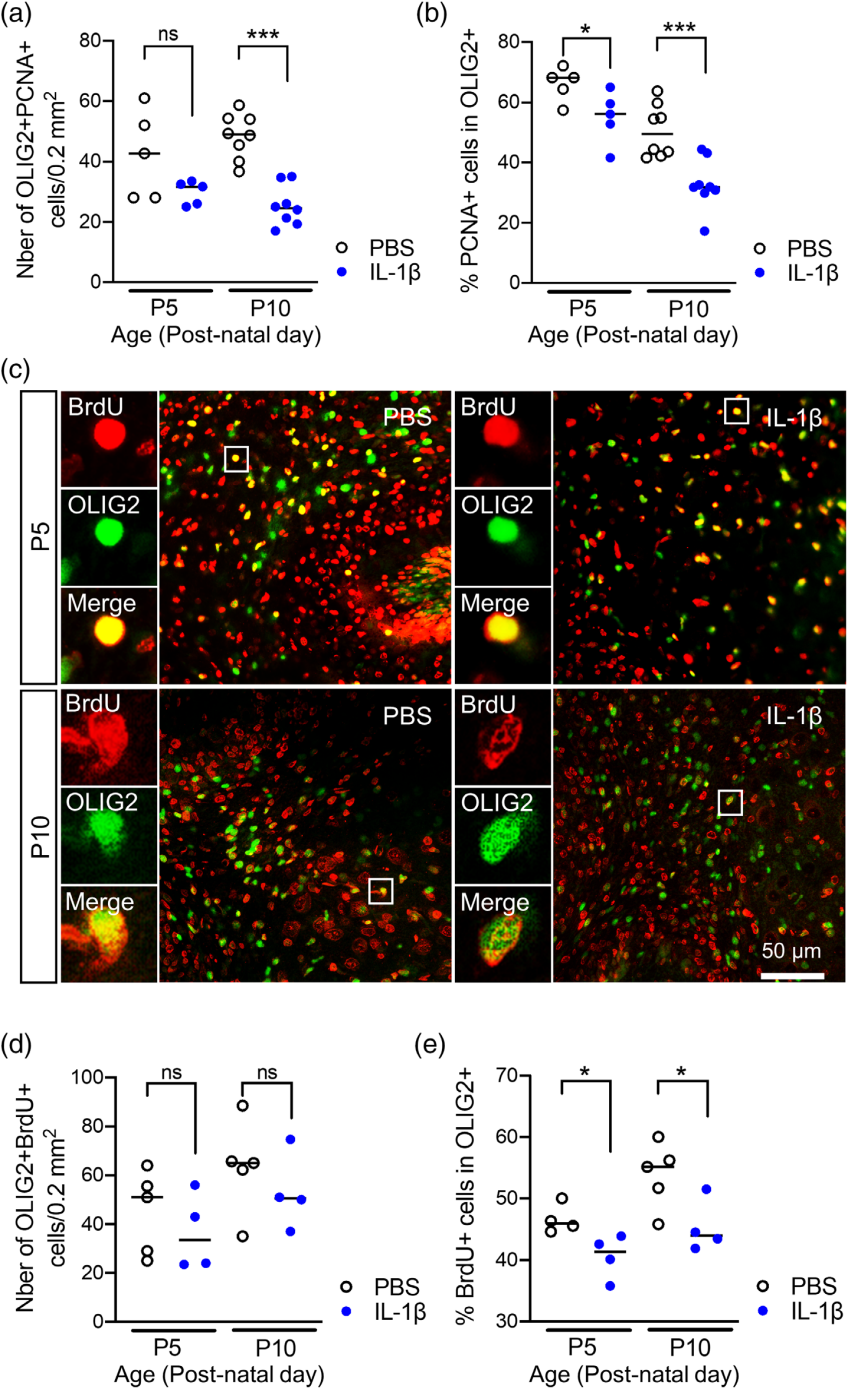
Impact of systemic inflammation on oligodendroglial proliferation. (a and b) Quantification of OLIG2+/PCNA+ cell numbers and OLIG2+/PCNA+ over OLIG2+ ratios in the cerebellum at P3 and P5 in PBS and IL‐1β‐exposed P5 pups. *p < .05, ***p < .001; *t*‐test. (c) Representative micrographs of proliferating (BRDU+, red) OLIG2+ (green) cells at P5 and P10 in cerebellar slices from IL‐1β‐exposed pups. Tissue section was selected by approximating 0.2–0.4 mm lateral position of Paxinos and Watson mouse brain atlas (2nd Edition, Academic Press, 2001). (d and e) Quantification of OLIG2+/BRDU+ cell number and OLIG2/BRDU+ over OLIG2+ ratios in the cerebellum at P5 and P10 in PBS and IL‐1β‐exposed P5 pups. Shapiro–Wilk test was used to verify normal distribution of each data set. Two‐tailed Student *t*‐tests were realized on data. *p < .05, ***p < .001. PBS, phosphate buffered saline

The rapid expansion of the cerebellar volume during development is characterized by a high proliferation of oligodendrocyte precursor cells (OPCs) and immature oligodendrocytes in the cerebellar white matter, while during further development, maturation is enhanced and numbers of mature, post‐mitotic oligodendrocytes increase (Baumann & Pham‐Dinh, 2001; Buffo & Rossi, 2013; Emery, 2010). Based on these data and having determined that there was a dysmaturation of oligodendrocytes, we next sought to determine if our macroscale structural MRI observations may also be explained by changes in oligodendrocyte *proliferation*. To measure proliferation of oligodendroglial lineage cells we first performed immunohistochemistry for OLIG2 in combination with proliferation marker PCNA. The absolute numbers of OLIG2+/PCNA+ cells in the cerebellum of IL‐1β‐exposed mice at postnatal age at P3 was not statistically significant; however at P5 were lower than in control littermates (median ± standard deviation; P3 PBS: 42.7 ± 14.6 vs. IL‐1β: 31.7 ± 3.94, *p* > .05, *n* = 5; P5 PBS: 49 ± 7.46 vs. IL‐1β: 24.5 ± 6.6, *p* < .001, *n* = 8; Figure 3a). As a ratio of all OLIG2+ oligodendroglial lineage cells in the cerebellar white matter (OLIG2+/PCNA+ over all OLIG2+ cells), the proportion of proliferating oligodendroglia is very high in control animals, and is significantly decreased at P3 and P5 in IL‐1β‐treated pups (median ± standard deviation; P3 PBS: 68 ± 5.6 vs. IL‐1β: 56 ± 8.8, *p* < .05, *n* = 5; P5 PBS: 49 ± 8.7 vs. IL‐1β: 32 ± 8.4, *p* < .001, *n* = 8; Figure 3b). To assess the cumulative proliferation from P1–P5, we delivered daily BrdU injections (P1–P4) and performed co‐labeling of OLIG2 and BrdU at P5 but also after 5‐days of recovery at P10 (Figure 3c–e). In contrast to the *snap‐shot in time* PCNA analysis (OLIG2+/PCNA+) which suggests a reduction caused by systemic inflammation exposure, the were no effects of systemic inflammation on the absolute cell counts for OLIG2+/BrdU+ cells (median ± standard deviation; P5 PBS: 51 ± 17 vs. IL‐1β: 34 ± 16, *p* > .05, *n* = 4–5; P10 PBS: 65 ± 19 vs. IL‐1β: 51 ± 16, *p* > .05, *n* = 4–5; Figure 3d). When expressed as a ratio to all OLIG2+ cells however, the OLIG2+/BrdU+ population converged with the PCNA data to reveal significantly reduced proportions of OLIG2+/PCNA+ cells in animals with systemic inflammation (median ± standard deviation; P5 PBS: 46 ± 2.4 vs. IL‐1β: 41 ± 1.8, *p* < .05, *n* = 4; P10 PBS: 55 ± 5.3 vs. IL‐1β: 44 ± 4.2, *p* < .05, *n* = 4–5; Figure 3e). 3D). Taken together, these results indicate that perinatal inflammation induced by systemic IL1β causes damage to the oligodendroglial cell population by reducing the proportion of oligodendroglial lineage cells that show proliferation activity. Given the fact that this finding persists at P10 beyond the acute phase of IL‐1β injection, maintained inflammatory responses, for example, by local microglia, may be assumed to cause or at least contribute to inhibited proliferation as a secondary mechanism.

### 3.4 Microglia were the predominant CD11B cell type in the cerebellum in control mice and in mice exposed to systemic inflammation

**FIGURE 4:**
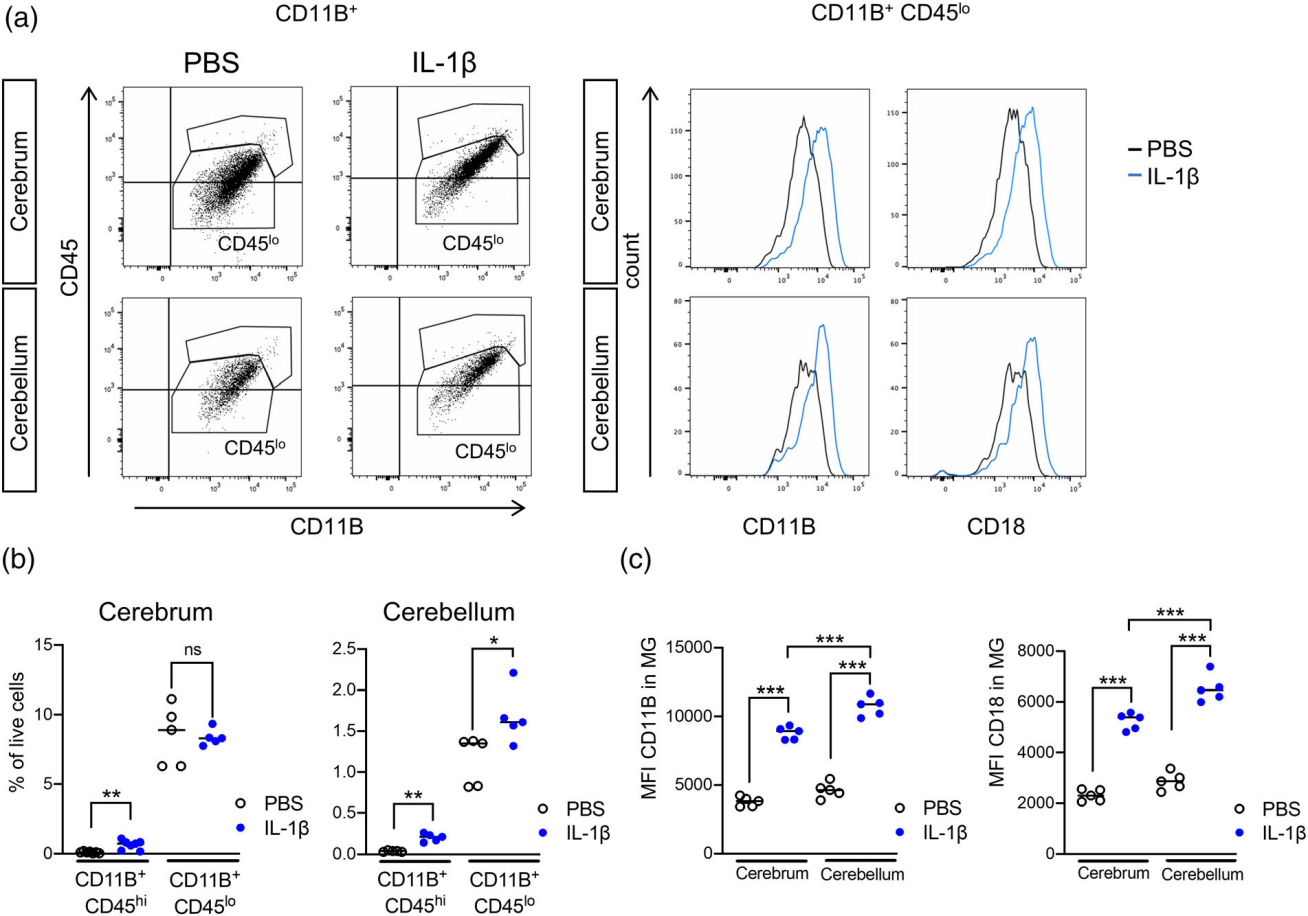
Impact of systemic inflammation on microglia. (a) Representative picture of flow cytometry gating strategies to analyze brain CD11B+ and CD11B+/CD45^int^ cells from the cerebrum and cerebellum of P5 IL‐1β‐treated and PBS treated mice and (b) the quantification of this data. (c) Quantification of dot plot analysis by flow cytometry of CD11B/CD18 MFI in CD11B+/CD45^int^ microglia in cerebellum and cerebrum from P5 IL‐1β‐treated and control mice. Shapiro–Wilk test was used to verify normal distribution of each data set. Two‐tailed Student *t*‐tests were realized on data. ++++ = cerebrum versus cerebellum or CD45^hi^ versus CD45^lo^, *p* < .001; **** = PBS versus IL‐1β, *p* < .0001; *** = PBS versus IL‐1β, *p* < .001; ** = PBS versus IL‐1β, *p* < .01. PBS, phosphate buffered saline

Our hypothesis based on prior information, is that microglia will drive the aforementioned white matter changes (Favrais et al., 2011; Krishnan et al., 2017; Van Steenwinckel et al., 2019). Thus having confirmed the presence of oligodendrocyte pathology in the cerebellum of IL‐1β‐exposed mice, we next explored these microglial characteristics in the cerebellum in order to compare with data from the cerebrum. We first undertook a characterization of CD11B+ expressing cells by FACS in the P5 cerebellum (Figure 4a). A high enrichment of CD11B+CD45^low^ microglia were found in the P5 cerebellum but also in the cerebrum (median ± standard deviation; cerebrum PBS: 8.9 ± 2.2 vs. IL‐1β: 8.3 ± 0.6, *p* > .05, *n* = 5; cerebellum PBS: 1.4 ± 0.3 vs. IL‐1β: 1.6 ± 0.33, *p* < .05, *n* = 5; Figure 4b). Notably, the percentage of CD11B+/CD45^int^ microglia is 6 times lower in the cerebellum than in the cerebrum at this age (Figure 4b). The numbers of CD11+/CD45^hi^ macrophages (Batiuk et al., 2020) in both brain structures increases slightly with IL‐1β injections (Figure 4b). This may reflect a modest myeloid cell infiltration, including neutrophils, monocytes and macrophages, as previously described in the forebrain by our group using this mouse model (Krishnan et al., 2017). A significant increase in the Mean Fluorescence Intensities (MFIs) of CD11B and CD18 were observed in CD11B+CD45^lo^ microglia in the cerebellum of IL‐1β exposed mice, which was also present in the cerebrum (median ± standard deviation; MFI CD11B cerebrum PBS: 3820 ± 350 vs. IL‐1β: 8925 ± 466, *p* < .001; MFI CD11B cerebellum PBS: 4627 ± 570 vs. IL‐1β: 10,898 ± 699, *p* < .001, MFI CD18 cerebrum PBS: 2305 ± 230 vs. IL‐1β: 5391 ± 331, *p* < .001; MFI CD18 cerebellum PBS: 2863 ± 351 vs. IL‐1β: 6466 ± 532, *p* < .001, *n* = 5; Figure 4c). Together these proteins form the phagocytic receptor CR3 (complement receptor 3), hence an increase may be suggestive of microglial activation, consistent with our prior observations in this model (Favrais et al., 2011; Van Steenwinckel et al., 2019). Importantly, the MFI of CD11B and CD18 in mice exposed to systemic inflammation is significantly higher (*p* < .001) in the cerebellum than in the cerebrum, suggesting a greater activation of cerebellar microglia compared with those in the cerebrum in response to systemic inflammation (Figure 4c). We next carried out immunostaining for CD3 in P10 cerebellar tissue to rule out a leucocyte/lymphocyte origin of the cells found after IL‐1β injections. Very few CD3+ cells (between 1 and 2 cells per total cerebellum section) were identified in the cerebellum of both experimental groups (Figure S5), suggesting that there is minimal invasion of blood‐derived cells in the cerebellum of the present model. Collectively, these data indicate that microglia are the predominant phagocytic cells in the cerebellum during the exposure to IL‐1β.

### 3.5 Systemic inflammation induces increases in microglial density and aberrant microglial morphology in the cerebellum

**FIGURE 5:**
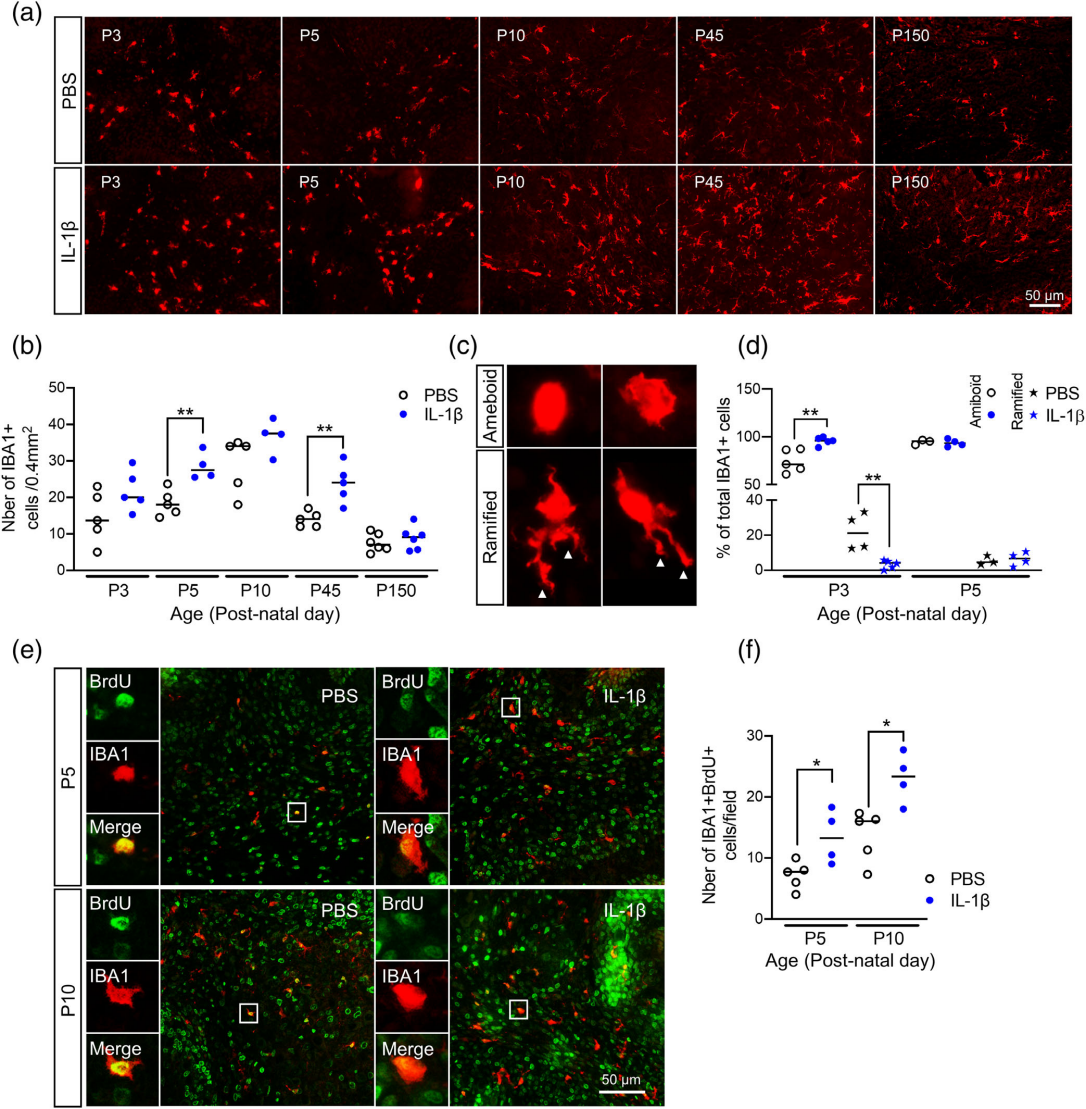
Impact of systemic inflammation on microglia activation and proliferation. (a and b) Representative micrographs of IBA1 at P3, P5, P10, P45, and P150 in cerebellar sections from PBS versus IL‐1β‐exposed pups (a) and corresponding quantification (b; *p < .05, **p < .01; Mann–Whitney *U* test). (c) Representative micrographs of amoeboid and ramified IBA1+ microglia observed at P3 and P5, with corresponding quantification (d, **p < .01, Mann–Whitney *U* test). (e and f) Representative micrographs of IBA1+ (green) and BRDU+ (red) cells at P5 and P10 in cerebellar sections from PBS versus IL‐1β‐exposed pups (e; Bar 50 μm) and corresponding quantification (f; *p < .05; Mann–Whitney *U* test). Tissue section was selected by approximating 0.2–0.4 mm lateral position of Paxinos and Watson mouse brain atlas (2nd Edition, Academic Press, 2001). PBS, phosphate buffered saline

We next analyzed IBA1+ microglial cell numbers at P3 and P5 during the exposure to systemic inflammatory challenge, at the juvenile age P45, and at the adult age of P150. In IL‐1β‐exposed mice, IBA1+ numbers were numerically higher at P3, P5 and even at P45 in comparison to controls although only the P5 and P45 time‐points were statistically significant (Figure 5a/b); there were no effects however at P10. Notably, at P150, IBA+ numbers were comparable in both groups (median ± standard deviation; P3 PBS: 14 ± 7.1 vs. IL‐1β: 20 ± 5.5, *p* = .11, *n* = 5; P5 PBS: 18 ± 3.6 vs. IL‐1β: 28 ± 3.8, *p* < .01, *n* = 4–5; P10 PBS: 34 ± 7.6 vs. IL‐1β: 38 ± 4.7, *p* = .12, *n* = 4–5; P45 PBS: 14 ± 2.1 vs. IL‐1β: 24 ± 5.3, *p* < .01, *n* = 5; P150 PBS: 7 ± 2.5 vs. IL‐1β: 9.1 ± 1.3, *p* = .45, *n* = 6; Figure 5b).

Microglia can be schematically described as having an amoeboid, ramified, or intermediate linked to distinct functional activities and although this is a simplification, it captures significant shifts in their functional state (Madore et al., 2013). An amoeboid morphology (representative image Figure 5c) is observed during (i) early brain development and it reflects the ongoing proliferation, migration and integration of these cells at that time (Dalmau et al., 1997; Ling, 1979), or (ii) during a pro‐typical pro‐inflammatory response wherein they are also often proliferating but performing immune functions. A ramified morphology (a surveying microglia), is observed in the uninjured adult brain and is characterized by small round soma with numerous processes (representative image Figure 5c). We therefore next assessed IBA1+ cell morphology as a crude proxy measure for microglia “state” in this model by applying a simplified method of classification as amoeboid for round IBA1+ cells and of ramified for IBA1+ cells with any process visible. At P3 and P5 in control (PBS exposed) pups, IBA1+ cells displayed amoeboid morphology; however, only at P3, the proportion of amoeboid IBA1+ microglia significantly increased in IL‐1β exposed animals (median ± standard deviation; P3 PBS: 71 ± 12 vs. IL‐1β: 96 ± 4.2, *p* < .01, *n* = 5; P5 PBS: 95 ± 2.6 vs. IL‐1β: 93 ± 3.8, *p* = .70, *n* = 3–4; Figure 5d). Conversely, in P3 PBS exposed mice, all IBA1+ cells were ramified, but IL‐1β exposure drastically reduced the number (median ± standard deviation; P3 PBS: 21 ± 11 vs. IL‐1β: 4 ± 2.4, *p* < .01, *n* = 4–5; Figure 5d). Since we also observed increased numbers and more amoeboid morphology of microglia, we hypothesized this would reflect increased proliferation. Indeed, there was a large increase in the number IBA1+/BRDU+ cells in the cerebellum at P5, at the end of IL‐1β exposure period, and also at P10, after 5 days of recovery, when compared to control pups (median ± standard deviation; P5 PBS: 7.7 ± 2.3 vs. IL‐1β: 13 ± 4.4, *p* < .05, *n* = 4–5; P10 PBS: 16 ± 4.2 vs. IL‐1β: 23 ± 4.1, *p* < .05, *n* = 4–5; Figure 5e/f). Collectively, these data suggest increased microglial proliferation, with a shift to an amoeboid morphology in the cerebellum that proceeds the development of oligodendrocyte pathology following systemic inflammation in mice, but normalizes over time.

### 3.6 The cerebellar microglial transcriptome in response to systemic inflammation had a specific IFN‐pathway signature

To better understand the spectrum of phenotypes of reactive microglia, information on gene expression and insights into signaling pathways are necessary. Whilst immunohistochemistry is invaluable for unbiased quantification of microglial number, density and morphology, this method is unsuitable for molecular phenotyping of microglial activation state. In our previous work in this mouse model we have provided evidence for specific transcriptomic changes in microglia linked to oligodendrocyte injury in the cerebrum (Favrais et al., 2011; Van Steenwinckel et al., 2019). Having confirmed the presence of microglial pathology in the cerebellum, we therefore measured the transcriptional profile of cerebellum microglia. In order to determine if microglia show comparable transcriptional changes in the cerebellum and the cerebrum, we analyzed the transcriptomic response of both populations of microglia to the inflammatory stimulus. Specifically, we isolated CD11B+ cells (primarily microglia, Figure 4) of the cerebrum and the cerebellum by magnetic cell sorting followed by transcriptome analysis with RNAseq at P5.

**FIGURE 6:**
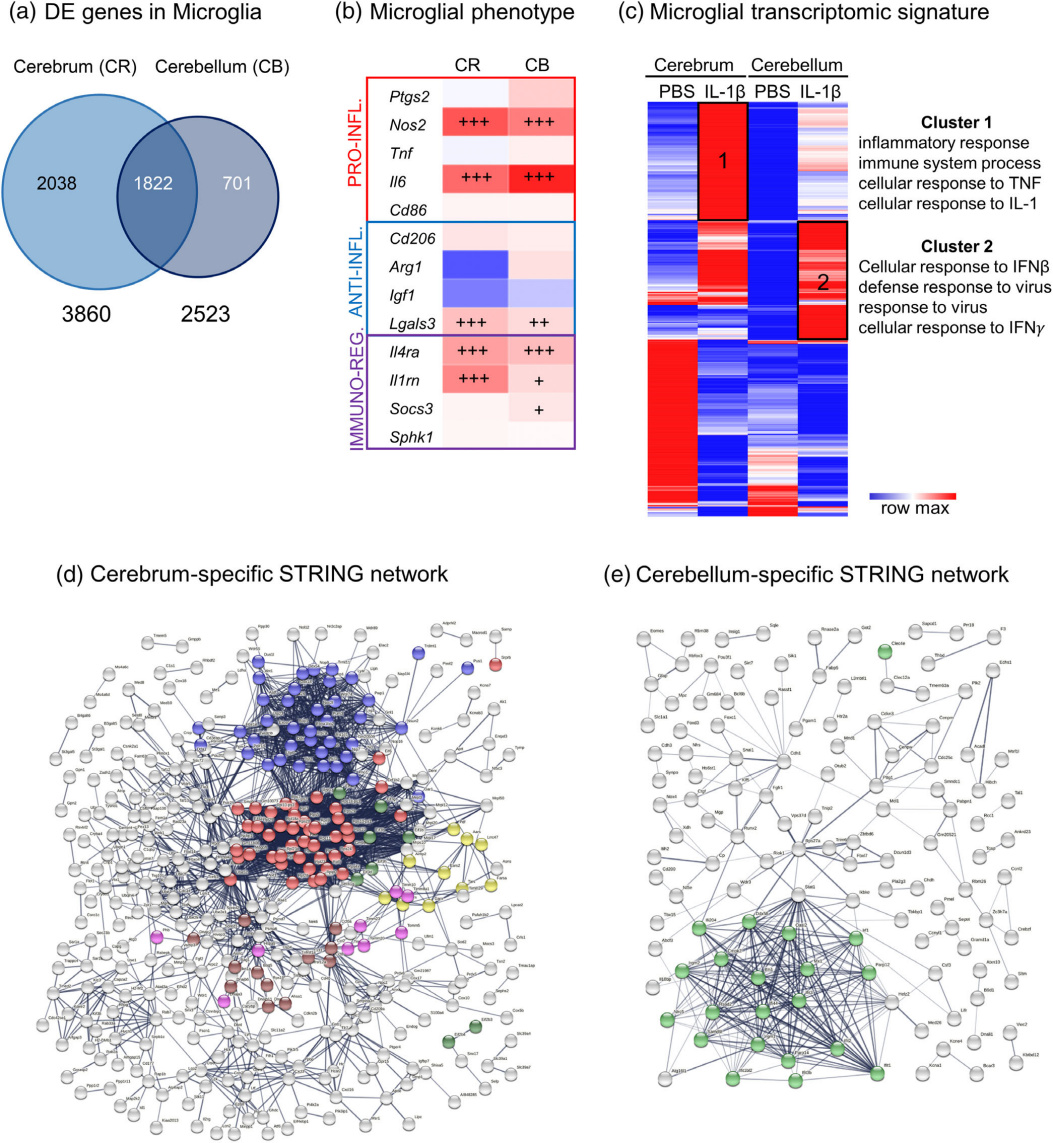
Region specific impact of systemic inflammation on the microglial transcriptome. (a) Venn diagram of Differentially Expressed (DE) genes in CD11B+ microglia in cerebrum and cerebellum from P5 IL‐1β‐treated and control mice. (b) mRNA expression of gene associated with different phenotypes in the cerebrum and cerebellum: pro‐inflammatory (PRO‐INFL.), anti‐inflammatory (ANTI‐INFL.) and immuno‐regulatory (IMMUNO‐REG.) markers (mRNA values in Table S4). (c) Heatmap of DE genes in cerebrum and cerebellum, and the Gene Ontology terms significantly associated to cluster 1 and 2. (d–e) Network representation from STRING showing cerebrum exclusive and cerebellum exclusive networks of predicted protein interactions. Colored dots indicate protein members of selected significantly (*q* < 0.05) enriched pathways. In (d), red, blue, yellow, green for translation processes (107/676 nodes); brown for heat shock responses (12/676); and pink for mitochondrial import processes (10/676). In (e), green for viral or interferon signaling (23/218 nodes). Viral or interferon signaling was not significantly enriched in (d)

Transcriptomic analysis revealed a higher number of differentially expressed (DE) genes induced by IL‐1β exposure in the cerebrum than in the cerebellum (3860 and 2523, respectively, with an adjusted *p* value <.05; Figure 6a, Table S3). Using data from the normalized read count matrix generated by the transcriptomic analysis (Table S4) we extracted data for analysis of microglial phenotype using previously validated gene expression markers (Chhor et al., 2013; Van Steenwinckel et al., 2019). This analysis revealed that in PBS‐exposed mice, CD11B+ microglia from the cerebellum expressed significantly different levels of expression across phenotype associated genes as compared to the cerebrum. For example, the gene expression counts for the pro‐inflammatory associated cytokine interleukin 6 (*Il6*), the anti‐inflammatory associated marker Arginase‐1 (*Arg1*) and the immunomodulatory marker Sphingosine kinase‐1 (*Sphk1*) were 35%, 90%, and 70% lower, respectively, in the cerebellum as compared to the cerebrum. By contrast, gene expression counts for the immunomodulatory marker interleukin 1 receptor antagonist (*Il1rn*) were 40% higher in the cerebellum than observed in the cerebrum. When comparing the response to IL‐1β, this revealed a similar microglial transcriptomic response in both the cerebellum and cerebrum, characterized by significant increases in pro‐inflammatory (inducible nitric oxide synthetase, *Nos2*, and *Il6* mRNA), immuno‐regulator markers (interleukin‐4 receptor antagonist, *Il4ra*, and *Il1rn* mRNA), and anti‐inflammatory galectin‐3 (*Lgals3* mRNA) mRNA markers, clearly indicating inflammatory activation in these microglia isolated from IL‐1β treated mice (Figure 6b, Table S4). In the cerebellum, there was a significant up‐regulation of the immuno‐regulator markers induced by IL‐1β (suppressor of cytokines 3, *Socs3* mRNA) (Figure 6b, Table S4).

To explore the regional differences and responses to inflammation we generated a heatmap from the DE genes induced by IL‐1β exposure in the cerebrum and/or in the cerebellum with a minimum of 2‐fold increase (LogFC > 1) or 2‐fold decrease (LogFC < 1), representing a total of 929 genes (Figure 6c, Table S5). One minus Pearson's correlation revealed region‐specific transcriptional identities of microglia from control (PBS‐treated) mice and those subjected to systemic inflammation (IL‐1β‐treated). Specifically, IL‐1β‐treatment revealed two relevant gene clusters with significant enrichment in Gene Ontology (GO) Biological Processes (BP). Cluster 1 comprised genes with a higher expression level in the cerebrum than in the cerebellum but with a similar fold change between PBS and IL‐1β in both structures. Cluster 2 comprised genes with a strong level of induction under IL‐1β in the cerebellum compared to the cerebrum. Functional enrichment analysis using DAVID 6.8 (Huang et al., 2007) revealed that Cluster 1 is enriched for GO terms related to inflammation, cell proliferation, extracellular signal‐regulated protein kinase (ERK) 1/2 and interferon (IFN)‐γ whilst Cluster 2 is enriched for GO terms related to response to virus, IFN‐β and IFN‐α (Figure 6c, Table S6). These data provide evidence to suggest that whilst the cerebrum and cerebellum microglia at P5 exist in distinct transcriptional states, their transcriptional programs also share common and distinct patterns of gene expression in response to IL‐1β, with a particular emphasis on increased activation of interferon signaling pathways in the cerebellum.

### 3.7 Cell‐location specific analysis confirmed a unique type‐II interferon response in cerebellar microglia

To explore further the specific differences between the response of the cerebrum and cerebellar microglia, we identified from our lists of DEG those genes that are exclusively altered in each population of microglia, as well as creating a list of commonly dysregulated genes. We explored these cell‐location‐exclusive and overlapping DEG lists using STRING and Wiki Pathways. STRING converted our gene data into known proteins and queried databases of established protein–protein interactions (PPI) to build networks (Table S7).

The PPI network for each of the three gene lists had significant (*p* < 1.0e^−16^) connectivity enrichment based on its size (adjusting for innate shell interactions) as shown in Figure 6d/e and Figure S6. Connectivity enrichment suggests biological interactions of the proteins compared with data sets generated from randomly selected proteins. For the shared gene list (Figure S6, Table S7), there was a significant enrichment (*q* < 0.05) in 71 known local network cluster groups, the vast majority (41/71) of which were directly related to ribosomes, proliferation, cytoskeleton, chromosomes, DNA replication or chromatin structure. Of note, 14/71 were related to mixed viral defense or inflammation, and of these 4/71 specifically to IFNB signaling. For the cerebrum specific network there was a significant enrichment (*q* < 0.05, Figure 6d) in 54 known local network cluster groups, the vast majority (39/54) of which were directly related to protein synthesis, especially ribosome function and none were related to viral responses. For the PPI network for cerebellum exclusive genes however, there was a significant enrichment (*q* < 0.05, Figure 6e) in 12 known local network cluster groups, all of which were related specifically to interferon or vial signaling.

We attempted to determine more precisely the differences in the specific DEG lists by querying the Wiki Pathway database (mouse). For the shared gene list, we identified five pathways significantly enriched (*q* < 0.05) all of which related to cell cycle, replication or cell survival (Table S8) but none to viral responses. Our cell type exclusive lists returned only one pathway for each cell type. For the cerebrum data, in agreement with our observations from our PPI network in STRING, we found enrichment in the Cytoplasmic Ribosomal Proteins (WP163) pathway (Table S8). Furthermore, we observed a significant enrichment in the Type II interferon signaling (IFNG and WP1253) pathway in the cerebellum microglia data, consistent with our STRING analysis (Table S8).

## 4 DISCUSSION

Despite the prevalence of cerebellar injury in preterm born infants (Basu et al., 2019; Bouyssi‐Kobar et al., 2016; Brossard‐Racine et al., 2015; Limperopoulos et al., 2007; Wu et al., 2020) and the links between these insults and poor functional outcomes (Shany et al., 2016; Tran et al., 2017), there are very few studies that have focused on the cerebellum in experimental models of preterm brain injury. Herein, we address this issue directly using an established mouse model of inflammation‐associated EoP. Our data provide evidence that induction of systemic inflammation leads to macroscale reductions in the volumes of the cerebellum gray and white matter, as measured by structural MRI, a clinically comparable technology. At the microscale, in line with our hypothesis, we demonstrate that these volumetric changes are preceded and accompanied by deficits in oligodendroglial proliferation, maturation, and myelination. Moreover, oligodendroglial pathology in this model is preceded and accompanied by increased microglial proliferation, characterized by amoeboid morphology and a specific transcriptional profile toward Type II interferon signaling.

Systemic inflammation in the fetal and neonatal period plays a key role in disturbance of brain development as shown clinically (Leviton et al., 2016, 2018; Patra et al., 2017) and supported by pre‐clinical models (Du et al., 2011; Favrais et al., 2011; Gussenhoven et al., 2018) (reviewed in Fleiss et al., 2015; Strunk et al., 2014). Prior studies in our validated mouse model of inflammation‐associated EoP provide evidence for disruption of white matter integrity in the cerebrum, characterized by elevated fractional anisotropy as measured by DTI (Favrais et al., 2011). To date, however, there has been no assessment of the neuroanatomical effects of systemic inflammation in this mouse model on either gray or white matter volumes. We therefore leveraged the whole brain coverage afforded by contrast enhanced ex vivo structural MRI to explore the pattern of regional brain volume changes at P15 and 60. We observed a reduction in mouse whole brain volume at P60 and in the absolute volumes (mm^3^) of several gray matter regions in the mouse cerebrum and cerebellum following perinatal IL‐1β exposure at both time‐points. Analysis of absolute volumes alone, however, cannot provide information as to whether these brain regions are smaller, but normally scaled, or if individual regions may be more, or less overgrown (abnormally scaled) (Clipperton‐Allen et al., 2019). This is relevant since smaller or larger, but normally scaled brains may not be pathological (Clipperton‐Allen et al., 2019; Mueller et al., 2021). Using relative brain volumes (% of total brain volume) as one means to address this question, we found no differences in the volumes of mouse brain regions located in the cerebrum between vehicle and IL‐1β‐exposed mice at either P15 or P60. By contrast, we observed a specific and consistent reduction in both the absolute and the relative volumes (% of total brain) of the cerebellar gray and white matter from P15 onwards. These data suggest that although absolute volumes of the cerebrum may be reduced by systemic inflammation, they appear to be normally scaled to total brain volume. By contrast, the cerebellum appears to be particularly sensitive to systemic inflammation during the perinatal period. These data complement the previous work by Favrias et al. to suggest that abnormal white matter microstructural changes in the mouse cerebrum following systemic inflammation do not appear to impact on the scaling of cerebrum gray matter, relative to total brain volume. Further work however incorporating longitudinal in vivo multi‐modal (structural and functional) MRI is required to confirm these observations and understand in depth how microstructural connectivity patterns relate not only to neuroanatomical changes in this model, but also to disruption of cognitive and emotional behaviors as previously reported elsewhere (Bokobza et al., 2022; Van Steenwinckel et al., 2019; Veerasammy et al., 2020).

Our structural MRI findings are therefore in good agreement with data from clinical studies of preterm infants, where exposure to inflammation is very common. Specifically, MRI‐detectable anomalies of the cerebellum are correlated with lower outcome scores in neurodevelopmental assessments (Rose et al., 2015). Furthermore, lower volumes of the cerebellum at term age equivalent are associated with reduced IQ and with poor language and motor performance at school age (Yanni et al., 2017). Of note, in a classical eyeblink conditioning test, former preterm infants showed cerebellar functional deficits even when they displayed mild or no cerebellar lesions (Tran et al., 2017) highlighting the importance of models with a mild phenotype. In this context, a post‐mortem study of the cerebellum of preterm infants without overt destructive brain damage revealed a significant cerebellar maldevelopment (Haldipur et al., 2011). In line with this view, our mouse model also produces no overt destructive lesions, cell death itself is very low and the primary injury is driven by dysmaturation (Favrais et al., 2011; Krishnan et al., 2017; Schang et al., 2014). Importantly, there are very few studies on the impact of perinatal systemic inflammation focused on the development of the cerebellum (Dean et al., 2009; McDougall et al., 2017) or including an analysis of this region (Du et al., 2011). Dean et al. exposed fetal sheep at 93–96 days gestational age (GA) once to 90 ng/kg of LPS and analyzed the brain 3 days later. Tolcos et al., exposed fetal sheep at 102 days GA once to the same dose of LPS (90 ng/kg) and analyzed the brain 9 days later. Interestingly, both groups reported cerebellar white matter injury without cell death, in agreement with our current work. In the model from Dean et al. (93–96 days GA, +3‐day analysis) however, there were no cerebellar gray matter changes, whilst Tolcos et al. (102 days GA sheep, +9‐day analysis) reported increased densities of mature neurons and the proliferative area of the external granule cell layer was increased. We have previously shown that the time between inflammatory exposure and analysis significantly impacts forebrain outcomes in a sheep model of inflammatory injury (Gussenhoven et al., 2018). Overall however, our data of decreased cerebellar gray matter volumes supports the previous findings of a vulnerability of the developing gray matter to inflammatory injury reported by Tolcos et al., and our findings of reduced molecular layer area in a mouse model of repeated exposure to a specific toll‐like receptor (TLR)‐2 agonist (Pam3CysSerLys4 and Pam3CSK4) (Du et al., 2011). Reductions in the volume of the cerebellum have also been found in a chronic inflammation model caused by glial fibrillary acidic protein (GFAP) driven transgenic IL‐6 production (Gyengesi et al., 2019). In this model, an elevated number of activated microglia was associated with lower cerebellar volumes at age 3 months, hence underlining the vulnerability of the developing cerebellum to inflammation.

Available data suggest that the density of microglia is lower in the developing cerebellum as compared to the developing forebrain (Stowell et al., 2018). Our FACS data in the present study support this previous report. Transient systemic inflammation however, caused a pronounced and sustained increase of the number of microglia in the cerebellum, characterized by higher number of amoeboid shaped microglia. These cerebellar data are in contrast to our previous data from the cerebrum in this inflammation model, where we did not observe a sustained change in microglia number (numbers returned to normal by P10) or robust change in morphology at any time point (Favrais et al., 2011; Krishnan et al., 2017; Van Steenwinckel et al., 2019). Morphology is often used as a surrogate indicator of the relative level of microglia “activation.” We have however, observed comparable levels of injury to oligodendrocytes in the cerebellum in this study and cerebrum in previous work from this model (Favrais et al., 2011; Schang et al., 2014) but this effect appears to be independent of microglia morphology, so further work is required to interpret this finding. The sustained alterations of microglia in the cerebellum could participate to the so‐called tertiary long‐lasting phase following an acute brain insult (Fleiss & Gressens, 2012), leading to a persisting set of changes that may sensitize to injury or prevent repair that might also underpin why whole brain volume changes became greater in magnitude with increasing age in this model. Altogether, these data support the hypothesis that microglia from different brain structures respond differently to inflammatory stimuli, shown previously for the gray and white matter in the cerebrum in adults (van der Poel et al., 2019).

As indicated above, microglia are drivers of injury and we have previously demonstrated a causal relationship between pro‐inflammatory microglia activation and hypomyelination in this model (Van Steenwinckel et al., 2019). Microglia also play an important role in the maturation and maintenance of oligodendrocyte populations (Hagemeyer et al., 2017; Miron et al., 2011). As such, our observations of increased numbers and altered activation of the cerebellar microglial after systemic inflammation are likely responsible for the oligodendrocyte dysmaturation and myelination deficit we observed via direct effects that injure these cells and possibly via disrupted development. This injury to oligodendrocytes was demonstrable as changes in oligodendrocyte maturation and myelin, and also reduced cerebellar white matter volumes on MRI. The important role of microglial activation for oligodendroglial and myelin damage has been elucidated by showing that reduction of microglial activation induced by microglial miRNA‐146b‐5p overexpression leads to significantly attenuated loss of oligodendroglial myelin production in mice with IL‐1β injections (Bokobza et al., 2022). Moreover, activation of microglial Wnt signaling pathway abolished microglial activation and at the same time restored myelination in the cerebrum of mice with IL‐1β exposure (Van Steenwinckel et al., 2019). These results of prior studies clarify a major role of microglial activation for oligodendroglial damage and myelin deficits in the cerebrum, which is likely to be true for oligodendroglial damage in the cerebellum, too although specific studies targeting microglial activation are required to confirm this. Based on the current study we also cannot exclude a contribution from other cell populations (i.e., astrocytes and NG2 glia), cell compartments (i.e., axons) and/or extracellular matrix protein abundance in the white matter volume changes. With regard to the reduction of gray matter volumes on MRI, further studies will be needed to validate our hypotheses of the presence of alterations in: (i) microglia mediated changes in synaptic and neurite pruning, as these are key functions of microglia (Lehrman et al., 2018; Paolicelli et al., 2011; Wolf et al., 2017), and; (ii) changes in the density of some neuronal subpopulations, as previously we have shown in this model changes in subclasses of interneurons (Stolp et al., 2019) and in neuronal‐associated gene expression in the cerebrum (Fleiss et al., 2015) and in another inflammatory model volumetric reductions in the molecular layer (Du et al., 2011).

As we investigated the transcriptomic profile of microglia in the cerebrum and the cerebellum, we noted that these cells have a common “activated” signature, characterized by an over‐representation of GO terms related to inflammation and cell proliferation. Analysis of the data across multiple approaches however, revealed that interferon (IFN) signaling, particularly type II IFN (IFN‐γ) signaling is specific to the cerebellum following IL‐1β exposure, which was not significantly observed in the cerebrum microglia. This specific response of cerebellar microglia may potentially contribute to long‐term neurodevelopmental sequelae of prematurity, including ASD. Indeed, clinical studies have shown that cerebellar abnormalities on MRI of preterm infants are a strong predictor of ASD in this high‐risk population, especially in the context of chorioamnionitis (Anblagan et al., 2016; Brossard‐Racine et al., 2015; Stoodley & Limperopoulos, 2016). A high activity of IFN‐γ has been found in children with ASD in comparison to healthy controls (Sweeten et al., 2004) as well as compared to children with non‐ASD developmental delay (Heuer et al., 2019). Treatment of neural progenitors derived from human induced pluripotent stem cells with IFN‐γ increased neurite outgrowth in these cells while inducing the expression of genes that have a role in schizophrenia and ASD, hence suggesting convergence of genetic and environmental factors (Warre‐Cornish et al., 2020). The specific overexpression of *Socs3* mRNA found in cerebellar microglia in the present study is in agreement with the fact that IFN‐induced signaling is regulated via SOCS proteins (Ivashkiv & Donlin, 2014). Type I IFN is known to mediate recruitment of inflammatory monocytes (Peralta Ramos et al., 2017) suggesting that it would be interesting to conduct an in‐depth study of myeloid cells infiltrated into the cerebellum in the present model. Altered type I IFN signaling in cerebellar microglia could also explain modification of microglial proliferation observed in the cerebellum in the present study as type I IFN is acknowledged to be an important regulator of microglial proliferation (Ben‐Yehuda et al., 2020).

The cerebellum is important for motor performance, memory, cognition, and emotion, and this region is vulnerable to injury in preterm born infants. As such, this project investigates the characteristics of cerebellar impairment after perinatal systemic inflammation to decipher the underlying cellular and molecular mechanisms for the long‐term goal of finding ways to improve outcomes in preterm infants. When compared to microglia of the cerebrum, our study provides novel and intriguing data in the cerebellum showing a strikingly sustained microglial activation (tertiary phase microglial dysfunction) combined with a specific microglial transcriptomic profile including altered IFN type I signaling following neonatal systemic inflammation. This unique pattern of cerebellar microglial activation might play a key role in the pathophysiology of cognitive and behavioral consequences of prematurity, making these cells a potential target for neuroprotection.

## AUTHOR CONTRIBUTIONS

Luisa Klein, Juliette Van Steenwinckel, Bobbi Fleiss, Christoph Bührer, Valerie Faivre, Dulcie A. Vousden, Anthony C. Vernon, Pierre Gressens, and Thomas Schmitz designed the research. Luisa Klein, Juliette Van Steenwinckel, Bobbi Fleiss, Till Scheuer, Valerie Faivre, Leslie Schwendimann, Zsolt Csaba, and Anthony C. Vernon performed the research. Sophie Lemoine and Corinne Blugeon performed the RNAseq. Luisa Klein, Juliette Van Steenwinckel, Bobbi Fleiss, Till Scheuer, Christoph Bührer, Valerie Faivre, Sophie Lemoine, CoB, Leslie Schwendimann, Zsolt Csaba, Dulcie A. Vousden, Anthony C. Vernon, Pierre Gressens, and Thomas Schmitz analyzed the data. Luisa Klein, Juliette Van Steenwinckel, Bobbi Fleiss, Anthony C. Vernon, Pierre Gressens, and Thomas Schmitz wrote the manuscript.

## CONFLICT OF INTEREST

The authors have declared that no conflicts of interest exist.

## Supporting information


**Figure S1** Timeline for injections and analyses. (A) Mice were administered IL‐1β (10 μg/kg/injection) or PBS twice a day for 4 days and once on day 5. Analyses were performed at various time points. (B) Animals were administered IL‐1β or PBS for 5 days as shown in (A). Additionally, BrdU was injected intraperitoneally. IHC, immunohistochemistry; PBS, phosphate buffered saline; PCR, polymerase chain reaction; WB, western blot.

**Figure S2** MRI segmentation. Example segmentation showing the registration of the DSURQE mouse brain MRI atlas to the minimum deformation template arising from the non‐linear MR image registration process. MR images are shown corresponding to approximately +2.96 to −8.12 mm from Bregma according to the mouse brain atlas of Paxinos and Watson (2nd Edition, Academic Press, 2001).

**Figure S3** Native Western blots. Western blotting of MAG and MBP of P10, P15, and P60 cerebellar tissues from PBS and IL‐1β‐exposed mice.

**Figure S4** Impact of systemic inflammation on oligodendrocyte cell death. (A) Quantification of OLIG2+ cells at P5 and P10 in cerebellum of PBS and IL‐1β‐exposed pups (***p < .001; *t*‐test). (B) Quantification of OLIG2+ CASP3+ double‐stained cells at P3, P5, and P10 in cerebellum of PBS and IL‐1β‐exposed pups (*p < .05; *t*‐test). (C) Representative micrographs of cerebellar slices showing OLIG2+ (green) and CASP3+ (green) cells in PBS and IL‐1β‐exposed pups (DAPI counterstaining in blue).

**Figure S5** Impact of systemic inflammation on blood‐borne CD3+ brain invasion. Representative micrographs of cerebellar slices (DAPI staining in blue) at P3 and P5 showing anecdotic CD3+ cells (red) in PBS and IL‐1β‐exposed pups.

**Figure S6** Network representation from STRING showing the predicted protein interactions network built with the shared gene list. Colored dots indicate protein members of selected significantly (*q* < 0.05) enriched pathways. Red = ribosome biogenesis, Blue = mixed anti‐viral defense, Magenta = DNA replication, Green = transmembrane receptor tyrosine kinase, yellow = kinetochores signal amplification (cell division).

**Table S1** List of antibodies and primers. (A) Primary antibodies for immunohistochemistry. (B) Secondary antibodies for immunohistochemistry.

**Table S2** Atlas‐based segmentation dataset, including absolute and relative volumes and statistical analysis of data from PBS and IL‐1β exposed mice.

**Table S3** Differentially expressed gene (DEG) lists from RNAseq analysis of CD11B+ microglia from the cerebrum and cerebellum.

**Table S4** mRNA values of phenotype associated genes used to create Figure 6b.

**Table S5** Gene list used to build the DEG heat map and analysis for clusters in Figure 6c.

**Table S6** Outputs from the cluster‐based gene analysis.

**Table S7** Specific gene lists used to build the predicted protein networks in STRING.

**Table S8** Wiki pathways outputs from the cerebellum only, cerebrum only and shared genes lists presenting the significantly enriched pathways.

## Data Availability

All new data are available from the authors on reasonable request.
