# Supplementary figures and images for: A unique cerebellar pattern of microglia activation in a mouse model of encephalopathy of prematurity
Source: Glia. 2022 May 17;70(9):1699–719. doi: 10.1002/glia.24190 (PMC9545095; doi:10.1002/glia.24190)

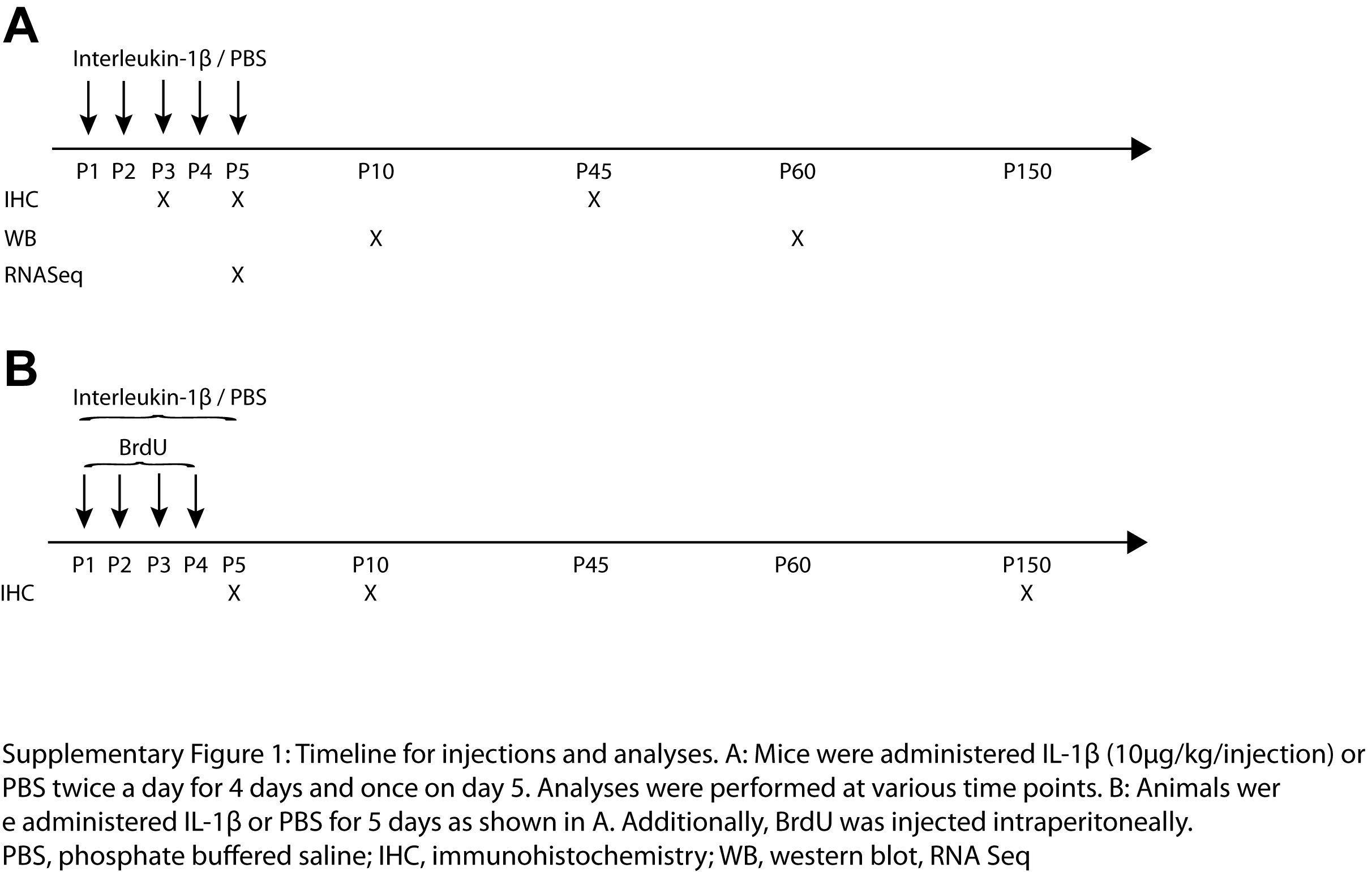

Supplement: Supplementary file 1 — Figure S1 Timeline for injections and analyses. (A) Mice were administered IL‐1β (10 μg/kg/injection) or PBS twice a day for 4 days and once on day 5. Analyses were performed at various time points. (B) Animals were administered IL‐1β or PBS for 5 days as shown in (A). Additionally, BrdU was injected intraperitoneally. IHC, immunohistochemistry; PBS, phosphate buffered saline; PCR, polymerase chain reaction; WB, western blot. [file GLIA-70-1699-s007.tif]

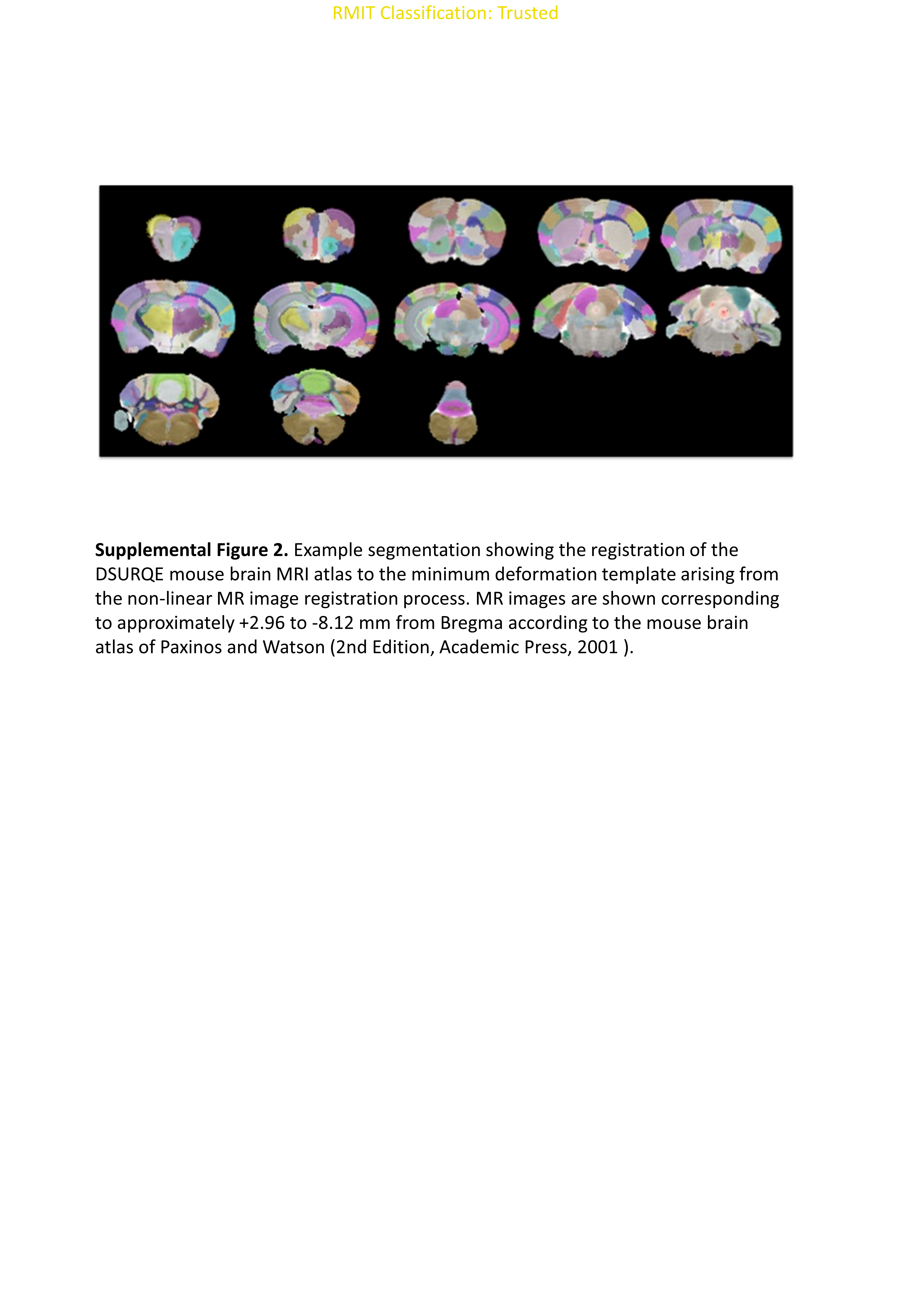

Supplement: Supplementary file 2 — Figure S2 MRI segmentation. Example segmentation showing the registration of the DSURQE mouse brain MRI atlas to the minimum deformation template arising from the non‐linear MR image registration process. MR images are shown corresponding to approximately +2.96 to −8.12 mm from Bregma according to the mouse brain atlas of Paxinos and Watson (2nd Edition, Academic Press, 2001). [file GLIA-70-1699-s004.zip › GLIA_24190_New supp fig 2_030922.tif]

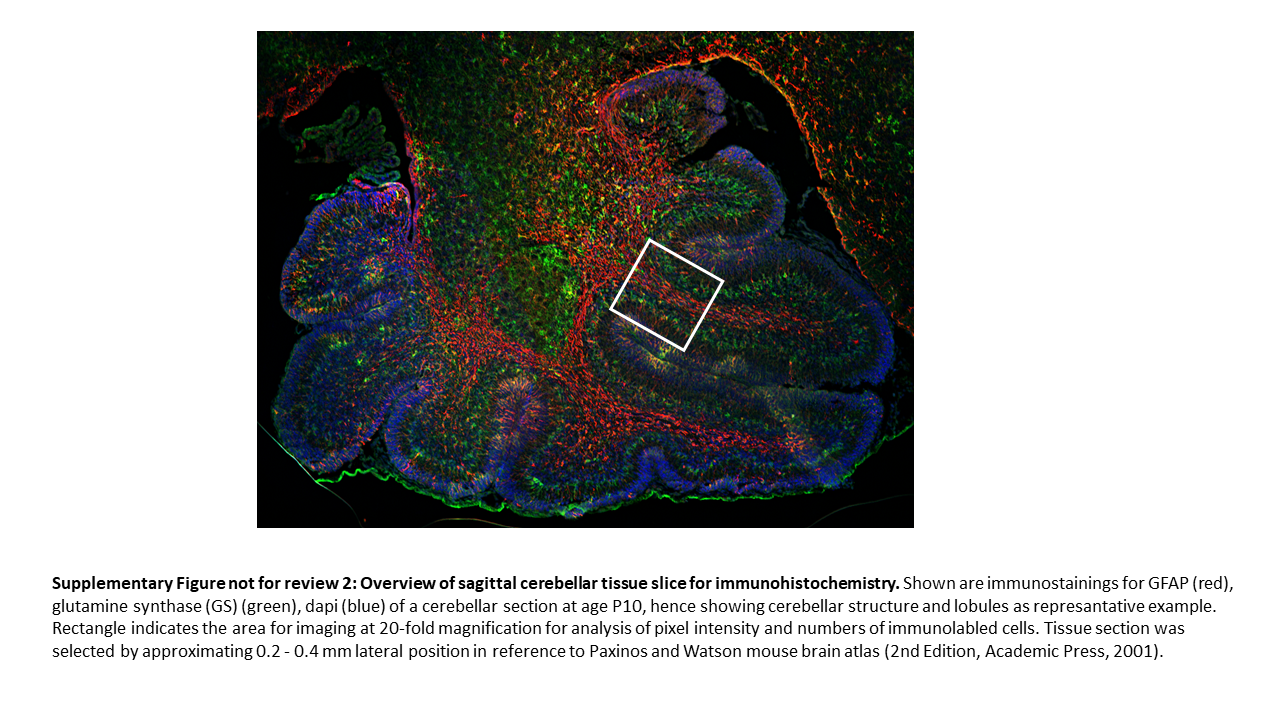

Supplement: Supplementary file 2 — Figure S2 MRI segmentation. Example segmentation showing the registration of the DSURQE mouse brain MRI atlas to the minimum deformation template arising from the non‐linear MR image registration process. MR images are shown corresponding to approximately +2.96 to −8.12 mm from Bregma according to the mouse brain atlas of Paxinos and Watson (2nd Edition, Academic Press, 2001). [file GLIA-70-1699-s004.zip › GLIA_24190_OVERVIEW_Cerebellum_astro 2.tif]

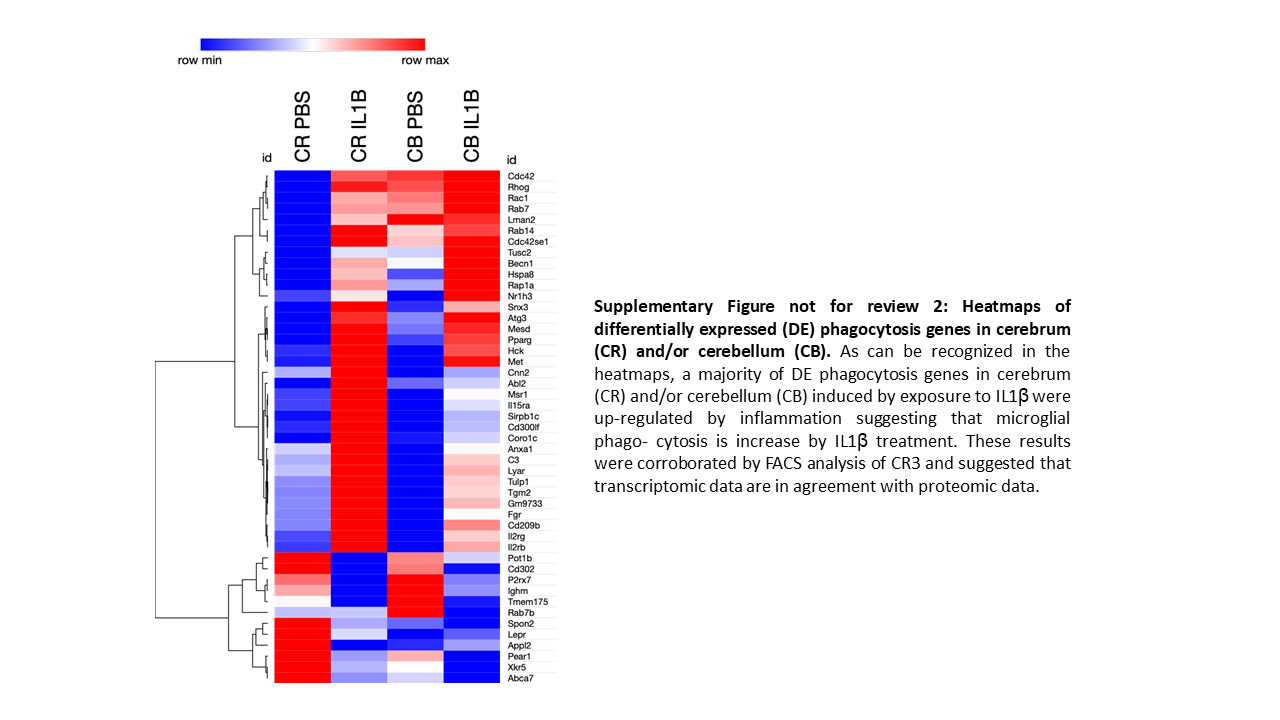

Supplement: Supplementary file 2 — Figure S2 MRI segmentation. Example segmentation showing the registration of the DSURQE mouse brain MRI atlas to the minimum deformation template arising from the non‐linear MR image registration process. MR images are shown corresponding to approximately +2.96 to −8.12 mm from Bregma according to the mouse brain atlas of Paxinos and Watson (2nd Edition, Academic Press, 2001). [file GLIA-70-1699-s004.zip › GLIA_24190_suppl figure not for review 2.tif]

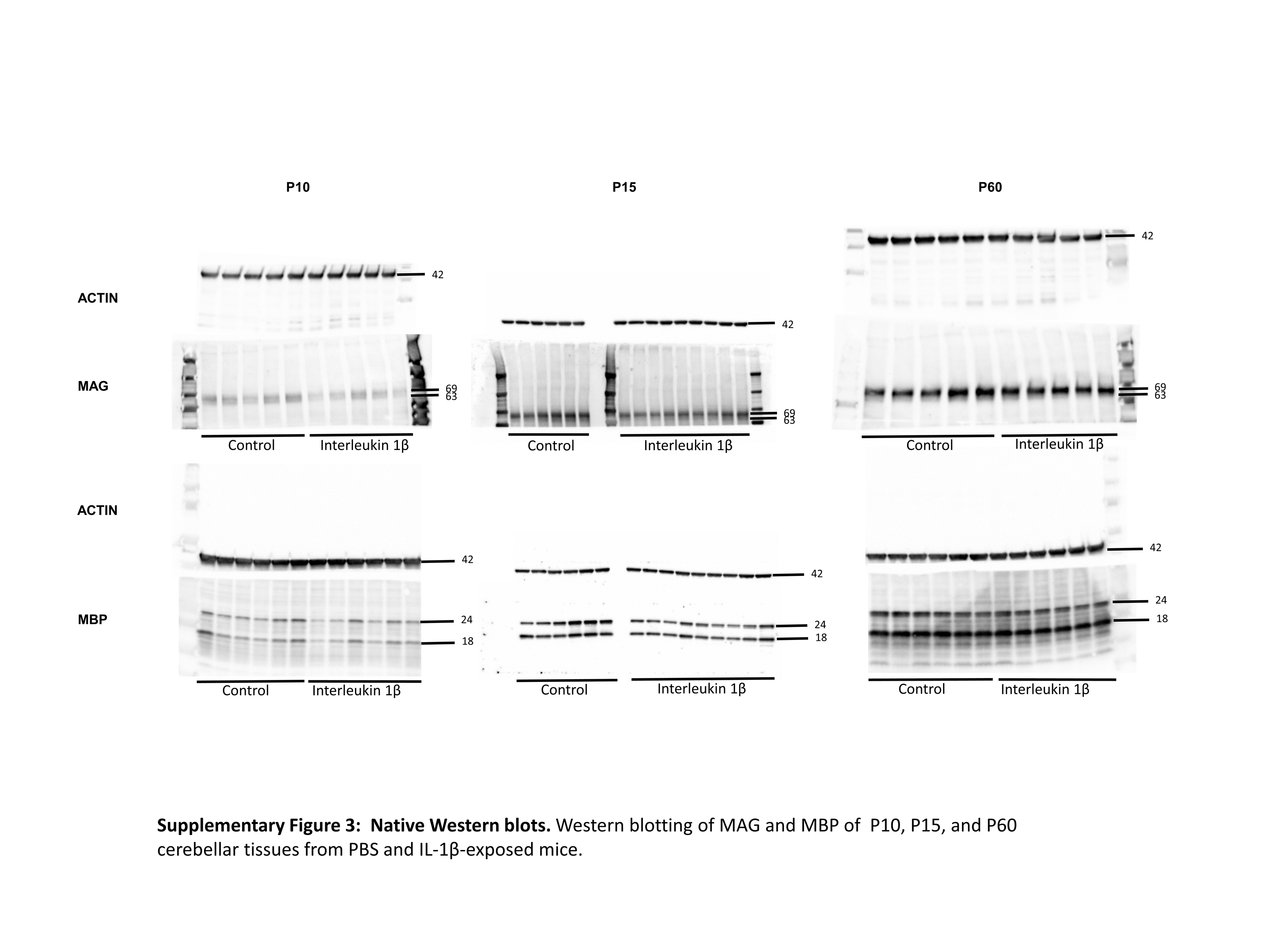

Supplement: Supplementary file 3 — Figure S3 Native Western blots. Western blotting of MAG and MBP of P10, P15, and P60 cerebellar tissues from PBS and IL‐1β‐exposed mice. [file GLIA-70-1699-s013.tif]

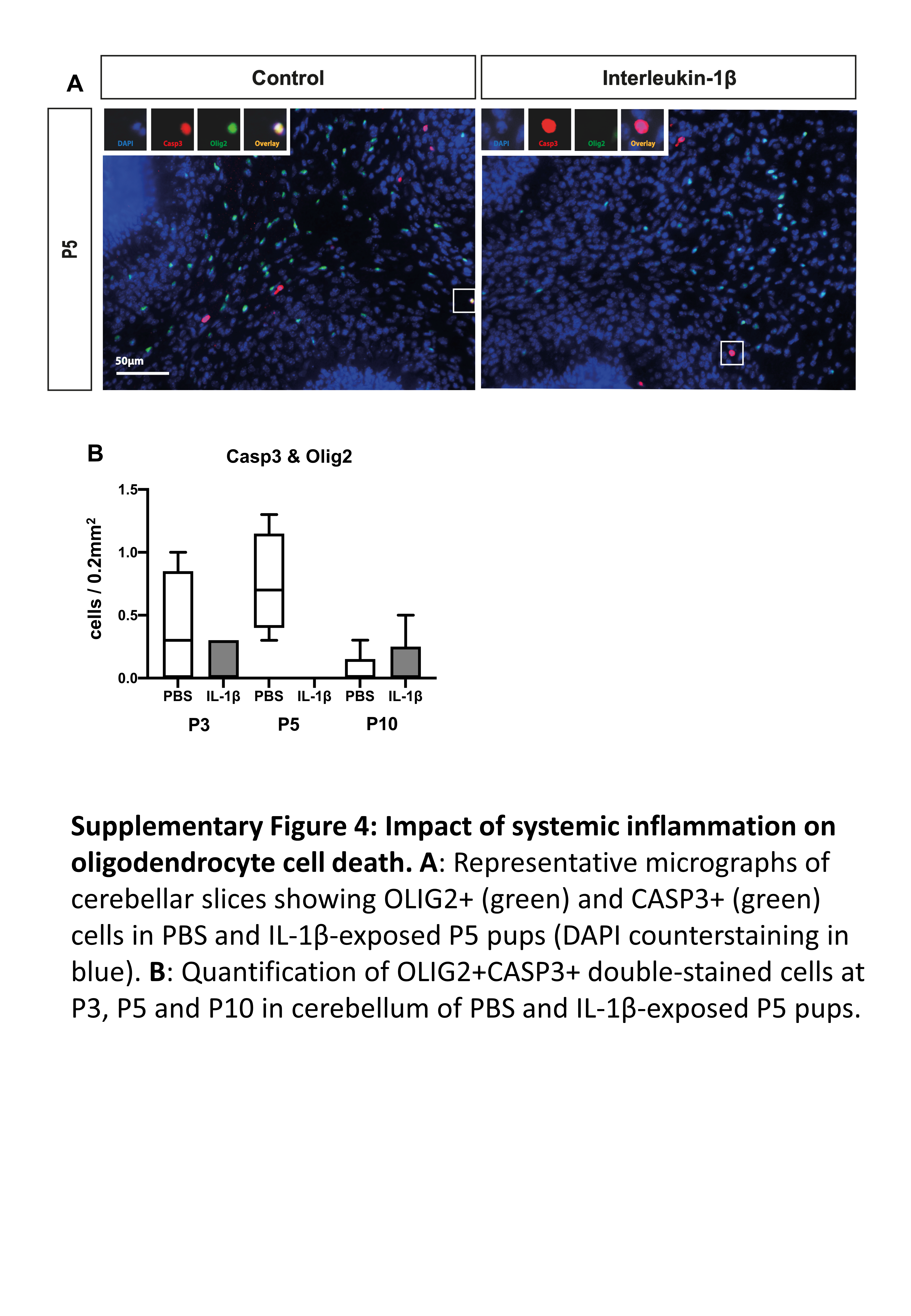

Supplement: Supplementary file 4 — Figure S4 Impact of systemic inflammation on oligodendrocyte cell death. (A) Quantification of OLIG2+ cells at P5 and P10 in cerebellum of PBS and IL‐1β‐exposed pups (***p < .001; t‐test). (B) Quantification of OLIG2+ CASP3+ double‐stained cells at P3, P5, and P10 in cerebellum of PBS and IL‐1β‐exposed pups (*p < .05; t‐test). (C) Representative micrographs of cerebellar slices showing OLIG2+ (green) and CASP3+ (green) cells in PBS and IL‐1β‐exposed pups (DAPI counterstaining in blue). [file GLIA-70-1699-s012.tif]

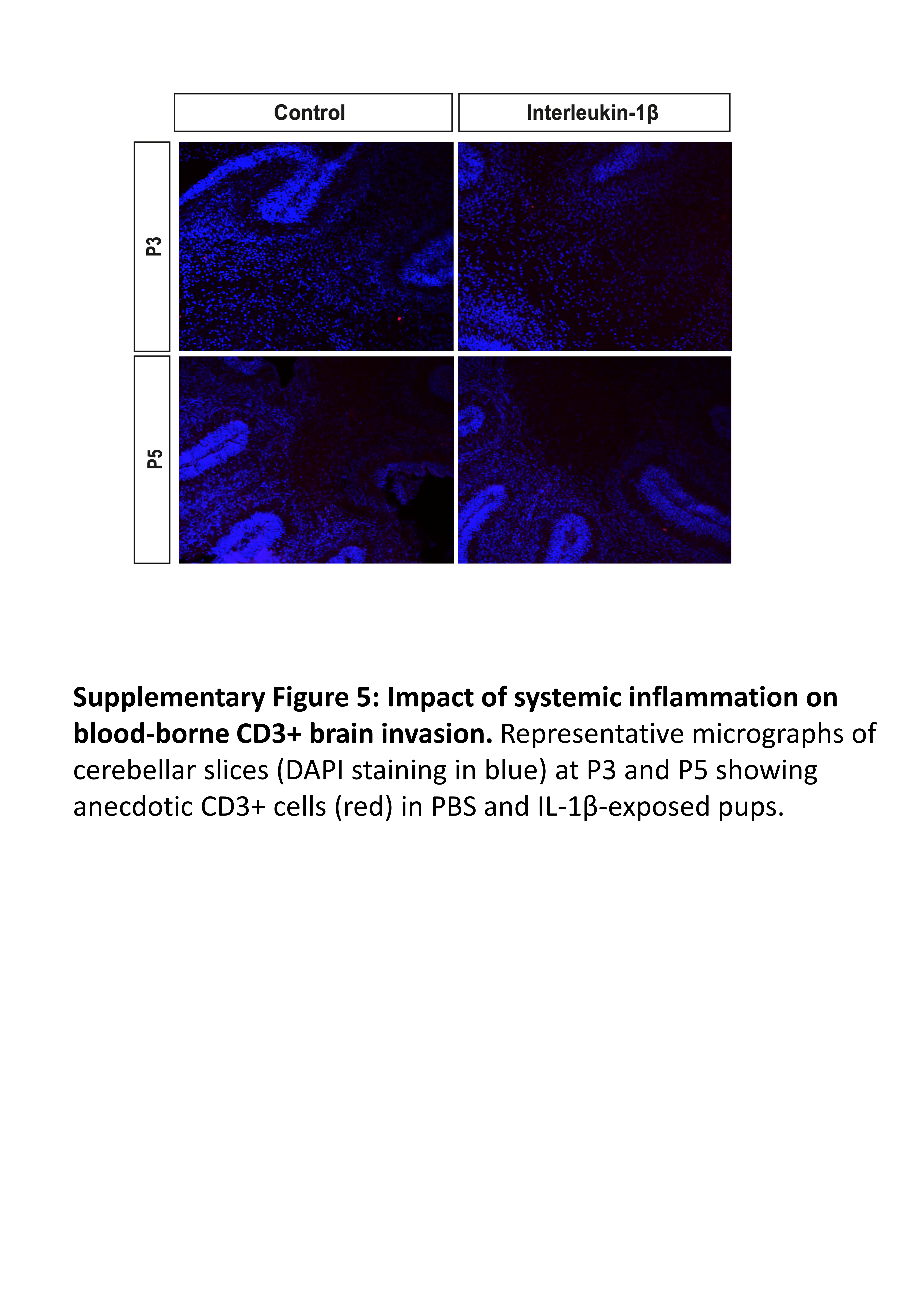

Supplement: Supplementary file 5 — Figure S5 Impact of systemic inflammation on blood‐borne CD3+ brain invasion. Representative micrographs of cerebellar slices (DAPI staining in blue) at P3 and P5 showing anecdotic CD3+ cells (red) in PBS and IL‐1β‐exposed pups. [file GLIA-70-1699-s005.tif]

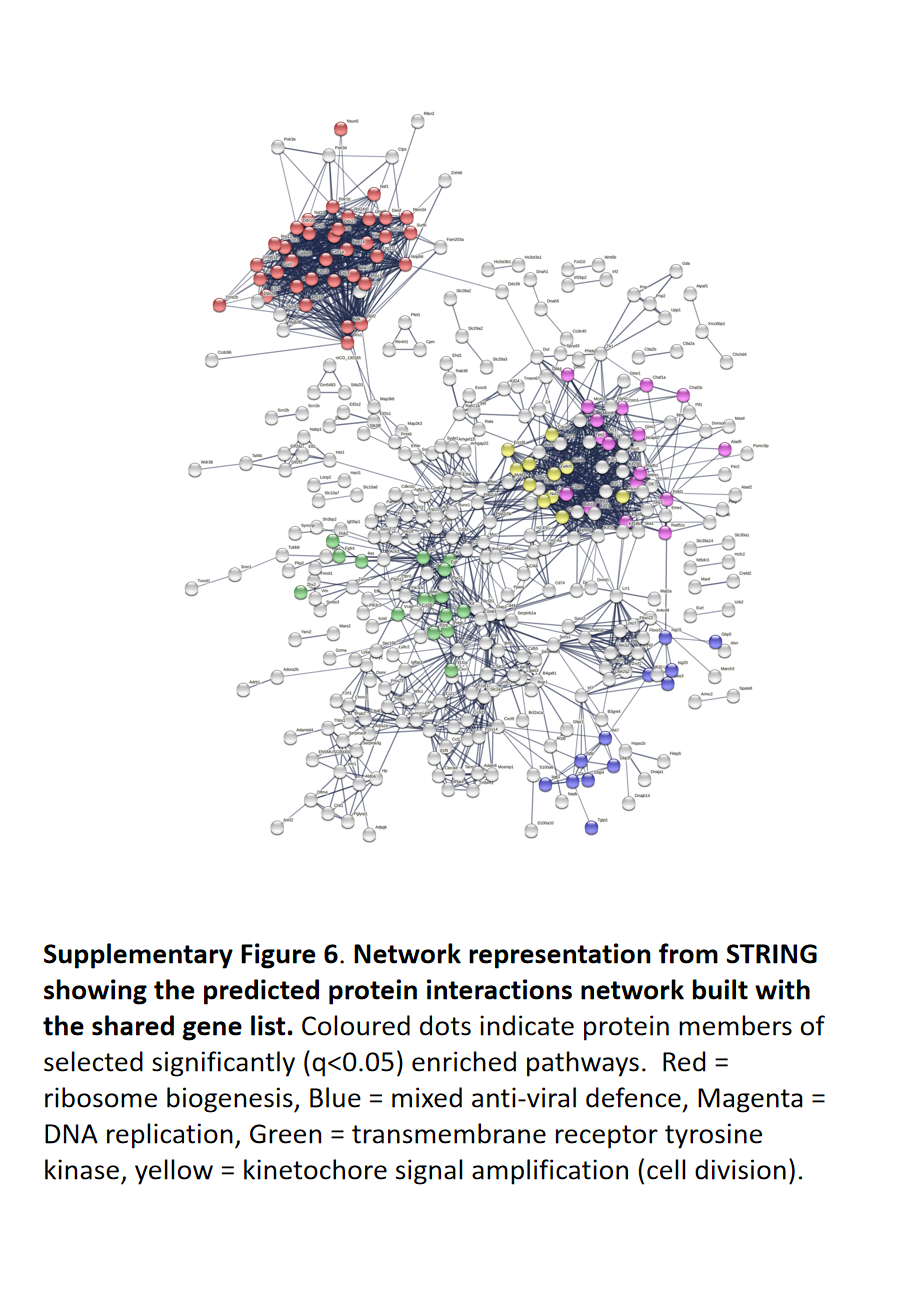

Supplement: Supplementary file 6 — Figure S6 Network representation from STRING showing the predicted protein interactions network built with the shared gene list. Colored dots indicate protein members of selected significantly (q < 0.05) enriched pathways. Red = ribosome biogenesis, Blue = mixed anti‐viral defense, Magenta = DNA replication, Green = transmembrane receptor tyrosine kinase, yellow = kinetochores signal amplification (cell division). [file GLIA-70-1699-s002.tif]
