# Supplementary material for: A unique cerebellar pattern of microglia activation in a mouse model of encephalopathy of prematurity
Source: Glia. 2022 May 17;70(9):1699–719. doi: 10.1002/glia.24190 (PMC9545095; doi:10.1002/glia.24190)
Supplement: Supplementary file 7 — Table S1 List of antibodies and primers. (A) Primary antibodies for immunohistochemistry. (B) Secondary antibodies for immunohistochemistry. [file GLIA-70-1699-s014.docx]

**Table 1A:** **Primary antibodies for immunohistochemistry**

| Antigen | Specification | Company | Number | Dilution | IT |
| --- | --- | --- | --- | --- | --- |
| BrdU | chicken anti BrdU | Abcam | ab92837 | 1:300 | 48h |
| BrdU | rabbit anti BrdU | Bioss | bs-0489R | 1:50 | 24h |
| Casp3 | rabbit anti Casp3 | Cell Signaling | 9664 | 1:200 | 24h |
| CC1 | mouse anti CC1 | Calbiochem | OP86 | 1:250 | 24h |
| CD3 | anti-mouse CD3, Alexa  Fluor^®^ 594 | BioLegend | AB_2553427 | 1:100 | 24h |
| Dapi |  | Sigma Aldrich | 32670 | 1:2000 | 10’ |
| Iba1 | rabbit anti Iba1 | Wako Chemicals | 019-19741 | 1:750 | 48h |
| MAG | mouse anti MAG | Abcam | ab89780 | 1:1000 | 24h |
| MOG | rabbit anti MOG | Abcam | ab32760 | 1:1500 | 24h |
| Olig1 | rabbit anti Olig1 | Millipore | AB15620 | 1:750 | 24h |
| Olig2 | mouse anti Olig2 | Abcam | ab56643 | 1:500 | 24h |
| PCNA | mouse anti PCNA | Abcam | ab29 | 1:1000 | 24h |
| PCNA | rabbit anti PCNA | Abcam | ab15497 | 1:500 | 24h |
| BrdU, Bromo-Desoxyuridine; Casp3, Cleaved Caspase 3; CC1, Anti-Adenomatous Polyposis Coli Clone 1; CD3, cluster of differentiation 3; Dapi = 4′,6-Diamidin-2-phenylindol; Iba1, Ionized Calcium-Binding Adapter Molecule 1; MAG, Myelin-Associated Glykoprotein; MOG, Myelin Oligodendrocyte Glycoprotein; Olig1, Oligodendrocyte Transcription Factor 1; Olig2, Oligodendrocyte Transcription Factor 2; PCNA, Proliferating Cell Nuclear Antigen; IT, incubation Time  Abcam, Cambridge, UK; BioLegend, San Diego, California, USA; Bioss, Boston, Massachusetts, USA; Calbiochem, Darmstadt, Germany; Cell Signaling Technology Europe, Frankfurt am Main, Germany; Milipore, Darmstadt, Germany; Sigma-Aldrich, Taufkirchen bei München, Germany; WAKO Chemicals, Neuss, Germany | | | | | |

**Table 1B: Secondary antibodies for immunohistochemistry**

| Antigen | Specification | Company | Number | Dilution | IT |
| --- | --- | --- | --- | --- | --- |
| chicken | goat anti chicken, Alexa Fluor^®^, 594nm | MP | A11042 | 1:200 | 1h |
| mouse | goat anti mouse, Alexa Fluor^®^, 488nm | MP | A11029 | 1:200 | 1h |
| mouse | goat anti mouse, Alexa Fluor^®^, 594nm | MP | A11032 | 1:200 | 1h |
| rabbit | goat anti rabbit, Alexa Fluor^®^, 488nm | Invitrogen | A11034 | 1:200 | 1h |
| rabbit | goat anti rabbit, Alexa Fluor^®^, 594nm | MP | A11037 | 1:200 | 1h |
| rabbit | donkey anti rabbit, Alexa Fluor^®^, 546nm | MP | A10040 | 1:200 | 1h |
| IT, incubation time; Invitrogen, Carlsbad, California, USA; MP = Molecular Probes, Waltham, Massachusetts, USA | | | | | |
